# A Multimodal Energy-Depletion Strategy for Cooperative Tumor Metabolism Regulation in Enhanced Cancer Therapy

**DOI:** 10.34133/bmr.0246

**Published:** 2025-11-17

**Authors:** Jingbo Ma, Kun Chen, Xiaoyong Zhang, Yanni Lou, Yunmeng Bai, Yinkwan Wong, Lei Zheng, Longying Li, YanWei Hu, Zhijie Li, Feng Qiu, Jigang Wang

**Affiliations:** ^1^ School of Chinese Materia Medica, Tianjin University of Traditional Chinese Medicine, Tianjin 301617, China.; ^2^ Center for Drug Research and Development, Guangdong Provincial Key Laboratory for Research and Evaluation of Pharmaceutical Preparations, Guangdong Pharmaceutical University, Guangzhou 510006, China.; ^3^ Guangdong Provincial Key Laboratory for Prevention and Control of Major Liver Diseases, Hepatology Unit and Department of Infectious Diseases, Nanfang Hospital, Southern Medical University, Guangzhou 510515, China.; ^4^ Department of Integrative Oncology, China-Japan Friendship Hospital, Beijing 100029, China.; ^5^ Department of Nephrology, Shenzhen Key Laboratory of Kidney Diseases, Guangdong Provincial Clinical Research Center for Geriatrics, Shenzhen Clinical Research Centre for Geriatrics, Shenzhen People’s Hospital (The Second Clinical Medical College, Jinan University, The First Affiliated Hospital, Southern University of Science and Technology), Shenzhen 518020, China.; ^6^ Guangdong Provincial Key Laboratory of New Drug Screening, School of Pharmaceutical Sciences, Southern Medical University, Guangzhou 510515, China.; ^7^ Department of Laboratory Medicine, Beijing Chao-Yang Hospital, Capital Medical University, Beijing, China.; ^8^ State Key Laboratory for Quality Ensurance and Sustainable Use of Dao-di Herbs, Artemisinin Research Center, and Institute of Chinese Materia Medica, China Academy of Chinese Medical Sciences, Beijing 100700, China.

## Abstract

Metabolic reprogramming represents a defining feature of the tumor microenvironment, driving both unchecked proliferation and therapeutic resistance. While conventional single-target metabolic therapies have demonstrated limited efficacy owing to the intrinsic adaptability of tumor cells, recent attention has turned toward natural herbal medicine. Combining broad, multilayered actions with low toxicity, they offer a promising way to modulate tumor metabolism and overcome current therapeutic limits. Herein, this work introduces an Artesunate/Icaritin (ART/ICA) hybrid nanoplatform derived from herbal medicine that employs a multimodal energy depletion strategy for malignant tumor therapy. Coadministration of ICA and ART in a nano-platform produces a mutually reinforcing effect that amplifies inhibition of glucose uptake, strengthens antiangiogenic activity, and intensifies mitochondrial dysfunction, overcoming the limitations of single-pathway interventions. The glutathione-responsive disulfide linkages in the nanomedicine enabled controlled, tumor-selective drug release, enhancing the therapeutic agents’ stability and bioavailability. In vitro mechanistic studies supported by RNA sequencing analyses and traditional molecular assays demonstrated that this multimodal approach effectively disrupted cellular energy homeostasis, induced apoptosis, and regulated key metabolic pathways. In vivo evaluations using various tumor models, including hepatocellular carcinoma transgenic mouse models, confirmed significantly enhanced antitumor efficacy, while subcutaneous tumor models showed a tumor inhibition rate exceeding 97%, far surpassing the effects of ART or ICA alone. Furthermore, flow cytometry analyses also confirmed that this strategy modulated the tumor microenvironment by enhancing the infiltration of cytotoxic CD8^+^ T cells and promoting dendritic cell maturation, while the incorporation of a CD47-targeting nanobody further strengthened immune activation and contributed to improved antitumor efficacy.

## Introduction

Malignant tumor remains one of the most prevalent and lethal malignancies worldwide, primarily attributed to its aggressive behavior and limited therapeutic options [1,2]. A defining feature of malignant tumor is the distinct reprogramming of cellular energy metabolism [3]. Compared with normal cells, tumor cells acquire metabolic alterations that not only fuel rapid proliferation but also enhance their resilience under hostile conditions [4,5]. The recognition that metabolic reprogramming plays a pivotal role in every stage of tumor development has led to the proposal of novel treatment modalities that target tumor energy pathways. Consequently, inhibiting or reshaping tumor energy metabolism has emerged as a promising approach to augment the efficacy and durability of anticancer therapies [6–8].

Tumor energy regulation can be examined and potentially modulated at several levels—ranging from the tissue microenvironment down to individual organelles. Firstly, at the tissue level, an extensive vascular network supplies nutrients and oxygen to actively dividing tumor cells [9]. Aberrant angiogenesis, driven primarily by factors such as vascular endothelial growth factor (VEGF), ensures that these cells receive abundant resources. Secondly, many tumors exhibit the well-known “Warburg effect” at the cellular level, favoring aerobic glycolysis over the more efficient oxidative phosphorylation [10]. Although glycolysis is less adenosine triphosphate (ATP)-efficient than mitochondrial respiration, its intermediate metabolites support biosynthesis and redox homeostasis, conferring a survival advantage. Meanwhile, at the organelle level, mitochondria act not only as powerhouses but also as crucial regulators of redox balance and apoptotic signaling [11]. Tumor cells modulate mitochondrial function to meet their ever-evolving metabolic and survival needs. These combined strategies demonstrate the adaptive flexibility of tumor metabolism and underscore the challenge of effectively disrupting these processes using a single intervention. Given these complexities, multiple intervention points have been proposed. At the tissue level, therapies that inhibit angiogenesis aim to deprive the tumor of critical nutrients and oxygen. Inhibitors of VEGF signaling, for instance, curtail excessive blood vessel formation [12–14]. At the cellular level, blocking glucose transporters (e.g., GLUT1) or inhibiting key glycolytic enzymes can disrupt the tumor’s preferred energy pathway [15–17]. At the organelle level, targeting mitochondrial function can compromise ATP generation and induce metabolic stress [18–20]. While these strategies highlight potential metabolic vulnerabilities, they often face challenges when employed independently. For example, antiangiogenic therapy alone can provoke hypoxia within tumor tissues, inadvertently selecting for more invasive, treatment-resistant phenotypes [21]. Similarly, impairing glucose uptake may drive tumor cells to switch to alternative nutrient sources such as amino acids or fatty acids, thereby blunting therapeutic impact [22,23]. Targeting mitochondria directly, though potent, can trigger compensatory pro-survival pathways and may enhance resistance to cell death [24].

These limitations reinforce the notion that a multimodal energy depletion strategy, capable of simultaneously influencing tumor metabolism at the tissue, cellular, and organelle levels, is anticipated to maintain the sustained therapeutic benefit of tumor energy-metabolism regulation. By systematically dismantling several energy pathways, multimodal approaches are better positioned to overcome metabolic plasticity and prevent the emergence of resistant clones. In this context, the use of traditional Chinese medicine (TCM) has attracted growing interest. Numerous TCM-derived compounds and extracts demonstrate multitargeted capabilities, such as suppressing aberrant angiogenesis, modulating glycolytic enzymes, and stabilizing mitochondrial functions [25–28]. Natural products like Icaritin (ICA) (from *Epimedium* species), Artemisinin, and their derivatives (from *Artemisia annua*) have shown promising antitumor effects and highlight the broad therapeutic potential of TCM in regulating tumor energy metabolism [27–31]. However, the clinical translation and therapeutic efficiency of TCM-based therapies are still limited by issues such as low water solubility, limited bioavailability, and the mild but multifaceted nature of their therapeutic actions [32]. While such attributes confer favorable safety profiles, they may also reduce efficacy when individual agents are used as monotherapies. Moreover, effective strategies for synergistically combining different TCM monomers or integrating TCM with conventional treatments remain insufficiently explored. Overcoming these challenges is imperative to leverage the ability of TCM to target multiple metabolic pathways, thereby facilitating the development of a promising comprehensive multimodal energy depletion strategy for malignancies. In aqueous solution, the amphiphilic nature of Pre-ART (Artesunate) enables spontaneous self-assembly into nanoparticles (NPs) via hydrophobic interactions between the hydrophobic alkane tails. This intrinsic property not only allows Pre-ART to function simultaneously as a drug carrier and a therapeutic agent but also facilitates effective encapsulation of additional hydrophobic compounds such as ICA, resulting in stable coassembled NPs. Such TCM-based self-assembled nanomaterials offer significant advantages, including enhanced aqueous dispersibility, improved bioavailability, and versatility in combinational strategies for multitargeted therapy.

**Fig. 1.**
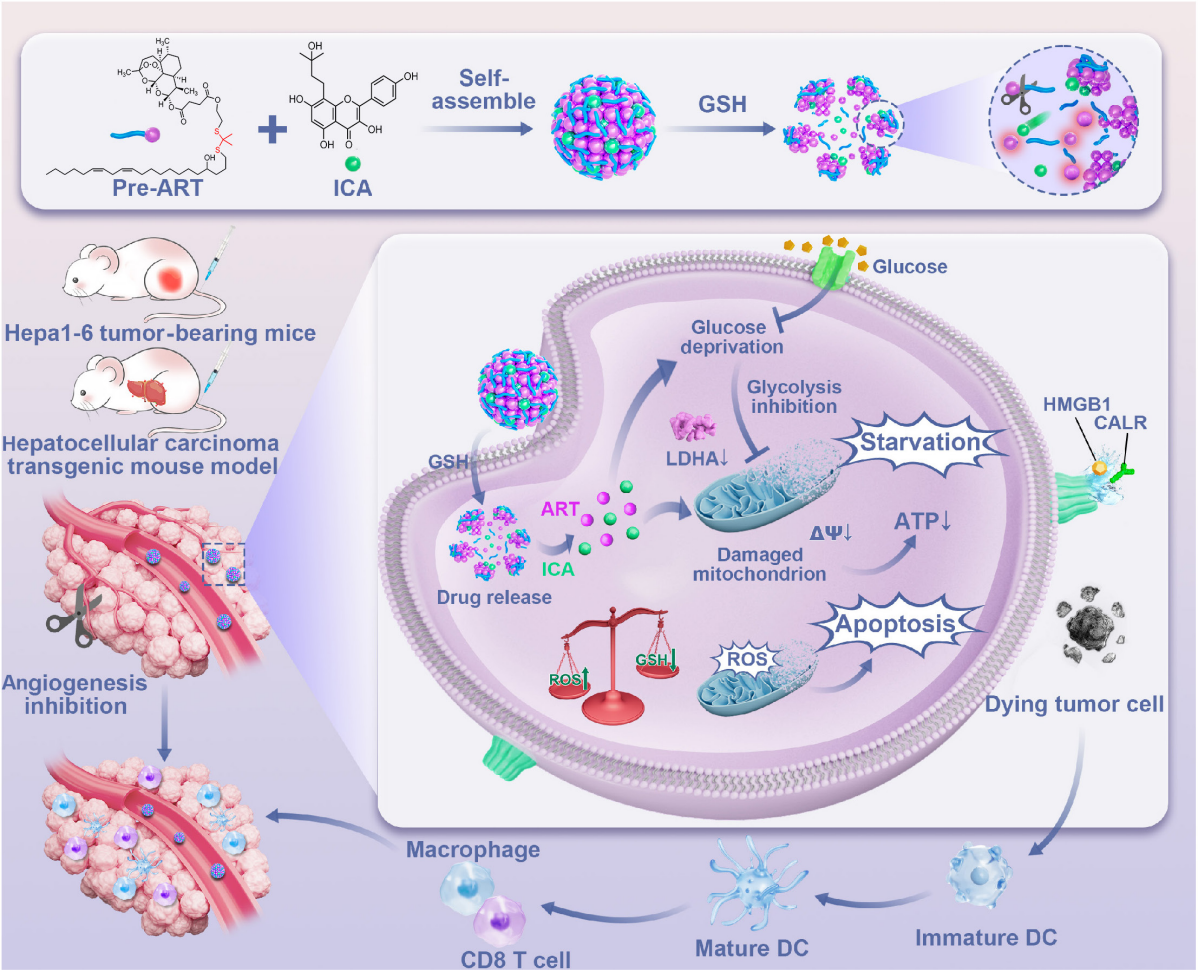
Schematic illustration of the mechanism by multimodal energy depletion strategy for tumor metabolism regulation in HCC treatment.

Thus, this study introduced a multimodal energy depletion strategy for tumor metabolism regulation in hepatocellular carcinoma (HCC) treatment, via the facile construction of an ART/ICA hybrid nanoplatform (as illustrated in Fig. 1). Codelivery of ICA and ART within the AI nanoplatform acts cooperatively to more powerfully suppress glucose uptake, augment antiangiogenic activity, and exacerbate mitochondrial dysfunction, thereby transcending the constraints of single-pathway therapies. The incorporation of glutathione (GSH)-responsive disulfide linkages provided a unique mechanism for controlled drug release, allowing the nanomedicine to deliver its therapeutic payload within the tumor microenvironment selectively. By improving stability and enhancing bioavailability, this platform ensured a more effective and localized antitumor response. Mechanistic in vitro studies, supported by advanced techniques such as the RNA sequencing and traditional molecular methods, revealed that the strategy induced substantial cellular energy imbalance, triggered apoptosis, and reprogrammed key metabolic pathways. In vivo results further corroborated its efficacy, with HCC transgenic mouse models showing pronounced tumor growth inhibition, and subcutaneous models achieving a tumor inhibition rate exceeding 97%, while ART or ICA alone demonstrated tumor inhibition rates of 58% and 62%, respectively. Additionally, flow cytometry (FCM) analyses indicated that this multimodal strategy reshaped the tumor microenvironment, boosting the infiltration of cytotoxic CD8^+^ T cells and enhancing dendritic cell (DC) maturation. The integration of a CD47-targeting nanobody amplified these effects by further activating immune pathways, resulting in a robust and sustained antitumor response. In summary, these findings highlight the potential of this multimodal energy depletion strategy as a promising avenue for improving the efficacy and durability of malignant tumor therapy.

## Materials and Methods

### Materials

Dichloromethane (DCM) was bought from Sigma-Aldrich. Deionized water was used in all experiments (Millipore). GSH was bought from MedChemExpress (MCE). Calcein AM and JC-1 Detection Kit were purchased from Beyotime Biotechnology. 4′,6-Diamidino-2-phenylindole (DAPI), Cell Counting Kit-8 (CCK8), and pyridine iodide (PI) were purchased from Solarbio Technology Co., Ltd.

### The preparation of ART derivative prodrugs (pre-ART)

To a solution of linoleic acid (1.0 g) and 1-ethyl-3-(3-dimethylaminopropyl)carbodiimide hydrochloride (EDCI) (815 mg) in DCM (25 ml) was added a solution of 4-dimethylaminopyridine (DMAP) (871 mg) in DCM (5 ml). The mixture was stirred at room temperature (RT) for 2 h. 2,2′-Disulfanediylbis(ethan-1-ol) (1.1 g) in DCM (10 ml) was added dropwise to the mixture. The mixture was then stirred at RT for 24 h. After the reaction was completed, the mixture was concentrated in vacuo and purified by silica gel column chromatography (EA/hexane, 1:40) to give CK-1 as a colorless solid (891 mg, 60% yield). ^1^H nuclear magnetic resonance (NMR) (400 MHz, CDCl_3_, ppm) δ 5.34 (h, *J* = 8.8 Hz, 4H), 4.35 (t, *J* = 6.4 Hz, 2H), 3.89 (t, *J* = 5.4 Hz, 2H), 2.91 (dd, *J* = 15.9, 6.6 Hz, 4H), 2.77 (s, 2H), 2.32 (t, *J* = 7.3 Hz, 2H), 2.04 (q, *J* = 6.6 Hz, 5H), 1.65 to 1.59 (m, 2H), 1.37 to 1.27 (m, 14H), and 0.89 (t, *J* = 6.3 Hz, 3H). ^13^C NMR (100 MHz, CDCl_3_, ppm) δ 173.71, 130.22, 130.02, 128.04, 127.88, 62.12, 60.18, 41.61, 37.02, 34.19, 31.51, 29.57, 29.33, 29.13, 29.08, 27.18, 27.17, 25.61, 24.86, 22.56, and 14.07.

To a solution of CK-1 (500 mg), artesunic acid (692 mg), and DMAP (220 mg) in DCM (10 ml) was added EDCI (106 mg). The mixture was stirred at RT for 24 h. After the reaction was completed, the mixture was added water and extracted with EA (3 × 10 ml). The combined organic extracts were washed with brine, dried over anhydrous Na_2_SO_4_, and concentrated to yield a crude product, which was purified by silica gel column chromatography (EA/hexane, 1:10) to give CK-2 as a yellow oil (588 mg, 49% yield). ^1^H NMR (400 MHz, CDCl_3_, ppm) δ 5.79 (d, *J* = 9.7 Hz, 1H), 5.43 (s, 1H), 5.34 (h, *J* = 8.2, 7.6 Hz, 4H), 4.34 (q, *J* = 8.6 Hz, 4H), 2.92 (t, *J* = 7.4 Hz, 4H), 2.79 to 2.55 (m, 7H), 2.44 to 2.23 (m, 3H), 2.03 (d, *J* = 9.1 Hz, 6H), 1.83 (d, *J* = 59.2 Hz, 3H), 1.66 to 1.48 (m, 5H), 1.43 (s, 3H), 1.33 (d, *J* = 18.2 Hz, 15H), 0.98 to 0.93 (m, 3H), and 0.91 to 0.82 (m, 6H). ^13^C NMR (100 MHz, CDCl_3_, ppm) δ 173.57, 171.87, 171.05, 130.20, 130.03, 128.02, 127.88, 104.45, 92.18, 91.48, 62.55, 62.03, 51.53, 45.20, 37.24, 36.98, 36.18, 34.15, 34.06, 31.77, 31.50, 29.58, 29.32, 29.15, 29.09, 28.77, 27.17, 25.94, 25.60, 24.86, 24.55, 22.55, 21.96, 20.19, 14.06, and 12.04. MS (ESI) [M + H]^+^: calcd for C_41_H_67_O_10_S_2_, 782.41; found, 783.64.

### The construction of ART derivatives/ICA nanoparticles

The ART derivatives/ICA nanoparticles (AI NPs) were self-assembled by combining the ART prodrug (Pre-ART) with ICA to form nanomedicines. Pre-ART (10 mg) and ICA (10 mg) were separately dissolved in 1 ml of dimethyl sulfoxide (DMSO) to prepare the stock solutions. The compounds were blended in a 9:1 (Pre-ART:ICA) mass ratio, followed by adding 100 μl of the mixed solution to 900 μl of water. The resulting mixture was subjected to dialysis in water for 6 h (3,000 Da) to remove any unencapsulated free drug.

### Basic characterization of AI NPs

The morphology and hydrodynamic size of the AI NPs were characterized by transmission electron microscopy (TEM) and dynamic light scattering (DLS). The stability of the AI NPs was assessed by exposing them to various environments, with their responsiveness evaluated through DLS and NMR. Additionally, ultraviolet–visible (UV–vis) spectrophotometry was employed to record the absorption spectra, confirming the successful encapsulation of the drug.

### Drug-releasing behaviors of AI NPs

The drug-loading and -releasing behaviors of AI NPs were evaluated by using high-performance liquid chromatography (HPLC) as follows: 1 ml of AI NPs was transferred to a dialysis bag (3,000 Da). The dialysis bag was placed in 50 ml of buffer (pure phosphate-buffered saline [PBS] with pH = 7.4 or PBS solution containing 1 mM GSH) and shaken in a shaker at a shaking speed of 100 rpm. At the appropriate time points (0, 0.5, 1, 2, 4, 8, 12, 24, 36, 48, and 60 h), 2 ml of dialysate samples were collected, and HPLC detected the content of ICA, and the same amount of fresh release buffer was supplemented.

### Cellular endocytosis and cytotoxicity studies

Then, Hepa1-6 or Huh1 cells were seeded in 6-well plates. After 24 h of culture, cells were coincubated with Cy5-labeled NPs for 0, 2, 4, and 6 h. After incubation, the cell nuclei were stained with DAPI and subsequently visualized using laser confocal microscopy. To quantitatively evaluate cellular uptake of AI NPs, FCM was performed on cells incubated with fluorescent NPs at varying time intervals.

The CCK8 method was used to detect the cytotoxicity of different drugs. In detail, Huh1 or Hepa1-6 cells in the logarithmic growth phase were inoculated in 96-well plates at 5 × 10^3^ cells per well and placed in an incubator overnight. On the second day, the cells were adherent, the supernatant was removed, and the complete medium containing different concentrations of ART, ICA, ART + ICA, and AI NPs was added. Seven concentration gradients (ART: 1.3, 2.5, 5, 10, 20, 40, and 80 μM; ICA: 0.13, 0.25, 0.5, 1, 2, 4, and 8 μM) were set for each group. After 24 h of drug action, CCK8 solution was added to each hole, and the OD_450_value of each hole was detected after shaking the orifice plate with a multifunctional microplate reader.

To further evaluate the toxicity of the drug, we performed live/dead cell analysis. Huh1 or Hepa1-6 cells in the logarithmic growth phase were seeded in a 24-well plate with $1\times 10^{4}$ cells per well and placed in an incubator overnight. On the second day, after the cells were adherent, the supernatant was removed and added to the complete medium containing ART, ICA, ART + ICA, and AI NPs (ART: 80 μM; ICA: 8 μM). After 24 h of drug action, the supernatant was taken and Calcein AM/PI detection working solution was added and photographed with a confocal microscope.

### Cell RNA sequencing

Total RNA was extracted from Hepa1-6 cells (*n* = 4 per group) using QIAzol Lysis Reagent (Qiagen) according to the manufacturer’s protocol. RNA concentration and purity were assessed with a NanoDrop 2000 spectrophotometer (Thermo Fisher Scientific), and integrity was evaluated using the Agilent 2100 Bioanalyzer. Samples with OD260/280 of 1.8 to 2.2, RIN ≥ 6.5, 28S:18S ≥ 1.0, and concentration ≥ 30 ng/μl were used for library construction. Libraries were prepared using the Illumina Stranded mRNA Prep, Ligation kit (Illumina), involving poly-A selection, fragmentation, and synthesis of first- and second-strand cDNA. After end repair, A-tailing, and adaptor ligation, fragments of ~370 to 420 bp were size-selected, PCR-amplified, and purified. Libraries were sequenced on the Illumina NovaSeq 6000 platform to generate 150-bp paired-end reads.

Raw sequencing reads in FASTQ format underwent quality control using fastp (version 0.22.0). Processed reads were then aligned to the mouse reference genome using HISAT2 (version 2.1.0), generating a quantitative gene expression matrix. Principal component analysis (PCA) was conducted using the *prcomp* function in R to assess overall sample variation and distribution. Differential gene expression analysis was performed using the DESeq2 R package (version 1.40.2), with significantly differentially expressed genes (DEGs) identified based on an adjusted *P* value < 0.05 and |fold change| > 1. Functional annotation of DEGs was conducted using the clusterProfiler R package (version 4.14.4), including Gene Ontology enrichment analysis for biological processes and Kyoto Encyclopedia of Genes and Genomes (KEGG) pathway enrichment analysis. Additionally, gene set enrichment analysis (GSEA) was employed to explore alterations in key gene sets between the 2 groups.

### Mitochondrial damage detection

Mitochondrial damage was assessed using fluorescence microscopy and FCM. Hepa1-6 cells were seeded in 6-well plates (5 × 10^5^ cells/well) and cultured for 24 h before treatment with ART, ICA, ART + ICA, or AI NPs. After 24 h of treatment, the medium was removed, and cells were washed twice with PBS. For fluorescence microscopy, cells were incubated with JC-1 (37 °C, 20 min), washed twice with JC-1 buffer, and imaged for red/green fluorescence. For FCM, cells were trypsinized into single-cell suspensions, washed with PBS, and stained with JC-1 (37 °C, 20 min). After washing with JC-1 buffer, green fluorescence was quantified.

### Detection of glycolysis pathway-related proteins

Hepa1-6 cells (2 × 10^5^ cells/well in 6-well plates) were treated with ART, ICA, ART + ICA or AI NPs for 24 h. Total protein was extracted using cell lysis buffer and quantified by bicinchoninic acid assay (BCA assay). Equal amounts of protein were separated by sodium dodecyl sulfate–polyacrylamide gel electrophoresis and transferred to polyvinylidene fluoride (PVDF) membranes. After blocking with 5% skim milk (2 h), membranes were incubated with primary antibodies (GLUT1, LDHA, and GAPDH) overnight at 4°C, followed by 1 h incubation with horseradish peroxidase-conjugated secondary antibodies at RT. Protein bands were visualized using enhanced chemiluminescence.

### Detection of intracellular lactate content

In this experiment, the intracellular lactate level was measured. Hepa1-6 cells were seeded in 6-well plates at a density of 1 × 10^6^ cells per well. After 24 h of culture, the cells were treated with ART, ICA, ART + ICA combination, and AI NPs, respectively. Following 24 h of drug treatment, the cells were washed twice with PBS, and the cell suspension was collected by trypsin digestion. After counting, 1 × 10^6^ cells were taken, and the intracellular lactate content was determined using a lactate assay kit.

### Angiogenesis experiment

Firstly, the matrix adhesive was slowly added to the 24-well plate, 250 μl per well to avoid bubbles. The MS1 cells treated with starvation for 24 h were inoculated in 24-well plates at a density of $5\times 10^{4}$ cells per well. At the same time, the cells were treated with ART, ICA, ART + ICA, and AI NPs, respectively. After 6 h, the supernatant was discarded and incubated with Calcein AM for 30 min and photographed.

### Immune cell activation in vitro

First, the maturation of DCs was detected using the mouse DC line DC 2.4. Then, Hepa1-6 cells were seeded in Transwell chambers at a density of $2\times 10^{5}$ cells per well. After 24 h of culture, the cells were treated with ART, ICA, ART + ICA, and AI NPs for 12 h. After that, a fresh medium was used instead of a drug-containing medium, DC 2.4 cells were inoculated into the lower hole of the Transwell, and Hepa1-6 cells without any drug treatment were used as the control group. After 24 h of incubation, DCs were collected for CD80 and CD86 antibody staining and FCM analysis.

### Construction of mouse tumor model

All animal experimental procedures were strictly performed in accordance with the Institutional Animal Care and Use Guidelines of Shenzhen People’s Hospital and were approved by the Institutional Animal Ethics Committee of Shenzhen People’s Hospital (Approval No. AUP-250423-LZJ-0456-1). In this study, Hepa1-6 and Huh1 tumor-bearing mouse models were employed to systematically evaluate the in vivo therapeutic efficacy of AI NPs. Hepa1-6 tumor-bearing mice model: logarithmic growth Hepa1-6 cells were prepared and injected subcutaneously into C57 mice according to the amount of $4\times 10^{6}$ cells per mouse. The tumor growth was observed and the tumor size was measured regularly. Follow-up studies can be performed when the tumor volume reaches 150 mm^3^.

Huh1 tumor-bearing mouse model: Huh1 cells with logarithmic growth were prepared and injected subcutaneously into NCG mice according to the amount of $5\times 10^{6}$ cells per mouse. The tumor growth was observed and the tumor size was measured regularly. Follow-up studies can be performed when the tumor volume reaches 150 mm^3^.

The HCC transgenic mice (Alb-tag^+^ mice) were kindly provided by Prof. Ruth Ganss from the University of Western Australia.

### Distribution of AI NPs in mice

The biodistribution of AI NPs in mice was monitored using an in vivo imaging system. ICG and ART prodrug were codissolved in DMSO to prepare NPs@ICG. When tumor volumes reached ~400 mm^3^, mice were intravenously injected with 200 μl of NPs@ICG or free ICG. Fluorescence signals were tracked at 2, 4, 8, 12, and 24 h postinjection. Following the final imaging time point, mice were sacrificed and major organs (heart, liver, spleen, lung, and kidney) along with tumors were collected for ex vivo fluorescence imaging.

### The antitumor effect of AI NPs in mice

The antitumor effect of AI NPs in mice was verified in different mouse models. First, Hepa1-6 tumor-bearing mice were divided into 5 groups (*n* = 5) for different administration treatments, namely, PBS, ART, ICA, ART + ICA, and AI NPs groups. ART 50 mg/kg and ICA 5 mg/kg were administered via tail vein every other day for 7 times. During the treatment, the body weight and tumor volume of the mice were monitored every day. At the end of the treatment, the mice were euthanized and the tumor tissues were weighed and photographed. Subsequently, the tumor tissues and major organs (including heart, liver, spleen, lung, and kidney) were embedded in paraffin sections for further hematoxylin and eosin (H&E) and related immunohistochemical staining. The treatment process of Huh1 tumor-bearing mice was consistent with the above. After 7 times of administration, the administration was stopped and the tumor size was continuously monitored. Mice with tumors growing to 1,500 mm^3^ or a weight loss of more than 20% were determined as the end point of the experiment and euthanized.

### Detection of immune response in the tumor microenvironment

When the tumor volume of Hepa1-6 tumor mice increased to about 150 mm^3^, ART, ICA, ART + ICA, and AI NPs were intravenously injected. On the next day after the last administration, the mice were euthanized and the tumor tissues were cut into pieces and digested in 5 ml of Dulbecco’s Modified Eagle Medium (DMEM) containing various enzymes at 37 °C for 50 min. The single-cell suspension was obtained with a 70-μm cell filter, and then the red blood cells in the cell suspension were lysed with red blood cell lysate. To detect T-cell surface markers, the cells were incubated with anti-mouse CD45-PE/CF594, anti-mouse CD3-FITC, anti-mouse CD8-APC/Cyanine7, anti-mouse CD11c-PE, anti-mouse MHCII-Percp/Cyanine5.5, and PI on ice for 1 h. To analyze the surface markers of macrophages, anti-mouse CD45-PE/CF594, anti-mouse F4/80-APC, anti-mouse CD86-APC/Cy7, and PI were incubated on ice for 1 h and stained.

### CD47 nanobody combined therapy

Hepa1-6 tumor-bearing mice were divided into 4 groups (*n* = 5) for different drug treatments, namely, PBS, CD47 nanobody, AI NPs, and AI NPs + CD47 nanobody group. AI NPs were administered intravenously every other day for a total of 3 times, and the therapeutic dose was set to ART 50 mg/kg and ICA 5 mg/kg. After the last administration of AI NPs, CD47 nanobody (100 μg/mouse) was intraperitoneally injected 2 times every other day. During the treatment, the body weight and tumor volume of the mice were monitored every day, and the mice with a tumor growth to 1,500 mm^3^ or a weight loss of more than 20% were determined as the end point of the experiment and euthanized.

### Statistical analysis

All statistical analyses were performed using GraphPad 9.0 (GraphPad Software Inc., USA). All data were presented as mean ± standard deviation (SD). For more than 2 groups of continuous variables, 1-way analysis of variance (ANOVA) or 2-way ANOVA were performed to determine the difference significance among groups. Differences between 2 groups of variables were compared and confirmed by 2-tailed Student’s *t* test. Survival curves were depicted using Kaplan–Meier’s method and compared by the log-rank test. In all cases, statistical significance was set as follows: *P < 0.05, **P < 0.01, ***P < 0.001, ****P < 0.0001, and not significant (n.s.).

## Results and Discussion

### The construction of AI NPs

**Fig. 2.**
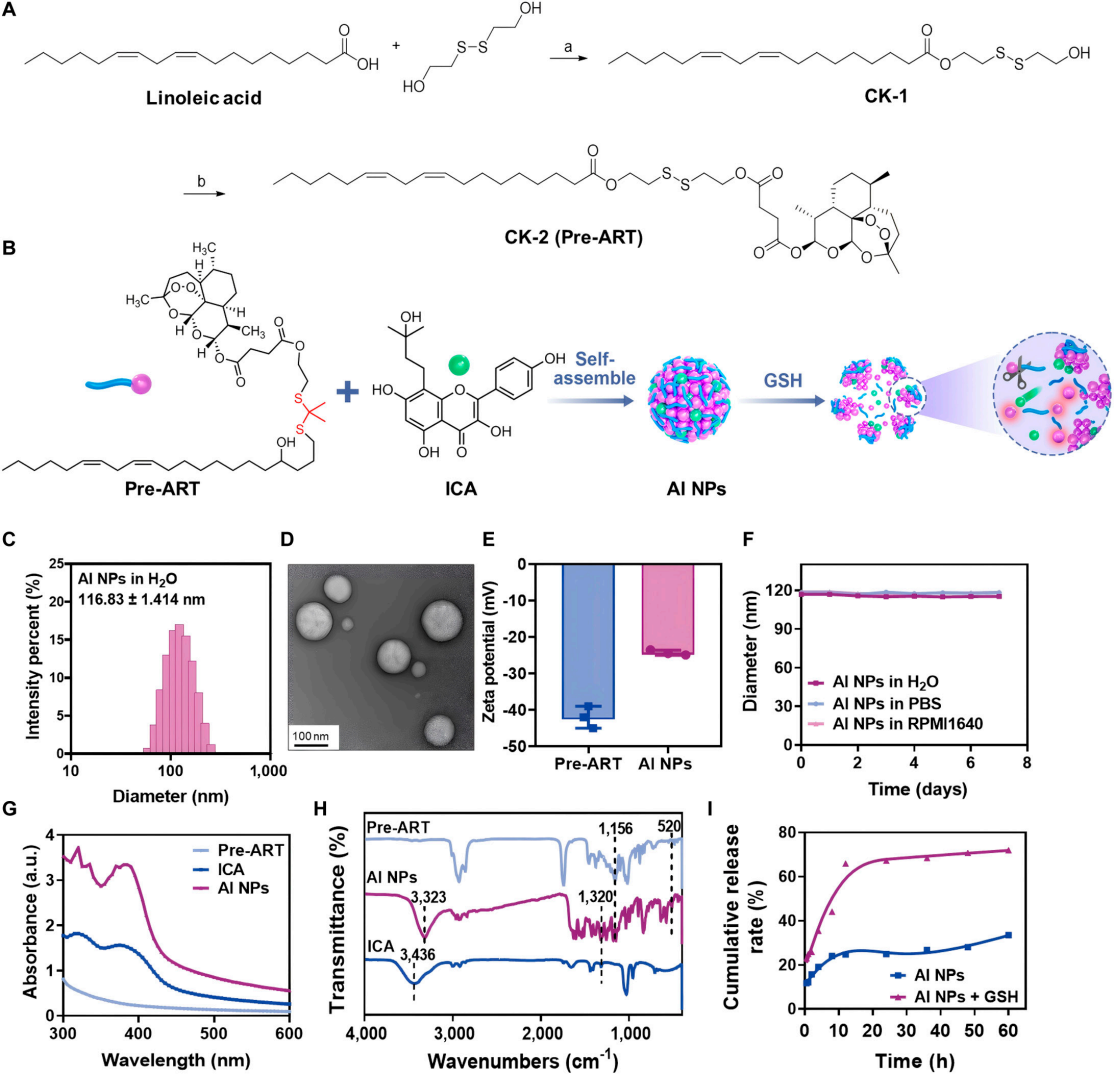
Characterization of the physicochemical properties of AI NPs. (A) Synthetic route of Pre-ART (CK-2). (B) Schematic illustration of the self-assembly of AI NPs. (C) Hydrodynamic size distribution of AI NPs. (D) TEM image of AI NPs. Scale bar: 100 nm. (E) Zeta potential of AI NPs in ddH_2_O. Data are presented as mean ± SD (*n* = 3). (F) Size stability of AI NPs in different solutions. (G) UV–vis absorption spectra of Pre-ART, ICA, and AI NPs. (H) FT-IR spectra of Pre-ART, ICA, and AI NPs. (I) ICA release profile of AI NPs in PBS (pH = 7.4) with 1 mM glutathione.

Briefly, the AI NPs were prepared by encapsulating the ICA with the ART derivatives through hydrophobic interactions of the alkyl chain molecules and π–π stacking interactions between the aromatic rings (as illustrated in Fig. 2B). The ART derivatives are synthesized by covalently linking oleic acid with an -S-S- group, which is subsequently conjugated to ART (Fig. 2A). The intermediate molecule CK-1 (covalently linking oleic acid with an -S-S- group) was characterized by ^1^H NMR and ^13^C NMR spectrum (Fig. S1), while the final product CK-2 (the ART derivative used in this study called Pre-ART) was confirmed through ^1^H NMR, ^13^C NMR spectrum, MS (ESI) spectra, and HPLC spectra (Figs. S2 to S4). The linker molecule containing the -S-S- group can undergo redox reactions with GSH, enabling the responsive cleavage of the linkage. This characteristic imparts GSH-responsive drug release capabilities to the ART derivatives, thereby facilitating the controlled release of drugs from AI NPs. The particle size distribution of AI NPs in aqueous solution, shown in Fig. 2C, reveals an average diameter of 116.83 ± 1.41 nm, indicating stable nanostructure formation. The particle size in PBS and RPMI 1640 culture medium remained around 118 nm (Fig. S5), suggesting that the AI NPs maintained a stable size in physiological solutions. The TEM image in Fig. 2D demonstrates the solid spherical morphology of the NPs. The zeta potentials of Pre-ART and AI NPs were measured to be −42.2 ± 3.02 mV and −23.43 ± 0.50 mV, respectively (Fig. 2E), suggesting the satisfactory charge stability of Pre-ART and AI NPs. To assess the hemocompatibility of AI NPs, we conducted hemolysis tests across a concentration gradient (Fig. S6). The observed hemolysis rates remained within acceptable thresholds at all tested concentrations, confirming their blood compatibility for potential in vivo administration. Then, the long-term aqueous particle stability of AI NPs was evaluated by DLS analysis in different media (water, PBS, and RPMI 1640) within 7 days (Fig. 2F). The obtained DLS data indicate that AI NPs remain relatively stable with diameters fluctuating between 110 and 120 nm in different media, suggesting their excellent colloidal stability for their subsequent biological applications. Next, the UV–vis absorption spectrum of Pre-ART exhibits noticeable fluctuations and absorption peaks in the 300 to 400 nm range (Fig. 2G), indicating the characteristic absorption features of the precursor drug. In contrast, the ICA spectrum is relatively smooth and stable, with minimal variation in absorbance across the 300 to 600 nm range. AI NPs, however, exhibit a pronounced absorbance peak in the 300 to 400 nm region, similar to that of Pre-ART, but with a broader absorption profile. This suggests the successful encapsulation of both Pre-ART and ICA within the NPs, further confirming the effective coloading of the drugs in AI NPs.

The infrared (IR) spectra in Fig. 2H show distinct absorption peaks at 1,156 and 520 cm^−1^ for Pre-ART, and at 3,436 cm^−1^ for ICA. These characteristic IR absorption bands appear near corresponding peaks in the AI NPs spectrum, further supporting the successful coencapsulation of Pre-ART and ICA within the NPs. Lastly, the cumulative drug release profiles for AI NPs and AI NPs + GSH over time, presented in Fig. 2I, demonstrate a significantly higher release rate of ICA in the presence of GSH. This indicates that GSH enhances the drug-releasing process, likely due to the interaction between GSH and the -S-S- groups in AI NPs, which alters their drug-releasing behaviors. These data collectively demonstrate the successful preparation of a multifunctional nanomedicine capable of GSH-responsive characteristics and codelivery of ART and ICA. This achievement lays a solid foundation for subsequent biological studies and anticancer applications.

### Cellular uptake and cytotoxicity of AI NPs in tumor cells in vitro

**Fig. 3.**
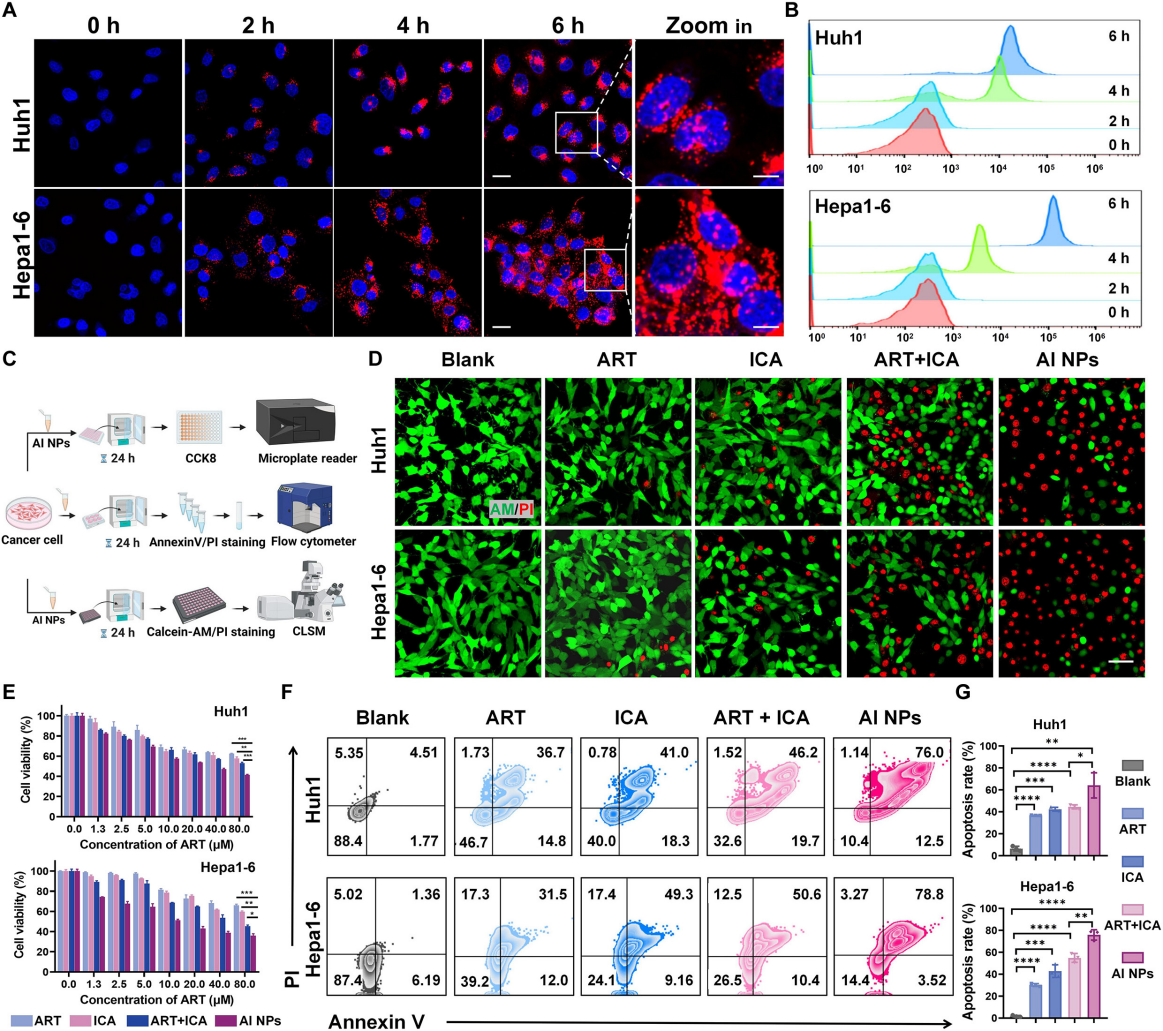
In vitro antitumor efficacy of AI NPs. (A) Confocal fluorescence images of cellular uptake of AI NPs in Huh1 and Hepa1-6 cells. Scale bars: 25 μm. Scale bars in the magnified images on the right: 10 μm. Blue: DAPI, red: Cy5-AI NPs. (B) Flow cytometry analysis of cellular uptake of AI NPs in Huh1 and Hepa1-6 cells. (C) Schematic diagram of in vitro cytotoxicity assay of Huh1 and Hepa1-6 cells treated with different formulations. (D) Live/dead staining, (E) cytotoxicity assay, (F) apoptosis detection, and (G) apoptosis rate of Huh1 and Hepa1-6 cells treated with different formulations. Data are presented as mean ± SD (*n* = 3). *P < 0.05; **P < 0.01; ***P < 0.001; ****P < 0.0001; one-way ANOVA.

Next, the basic biological properties of AI NPs, including their cellular uptake efficiency and cytotoxicity, were initially evaluated using confocal laser scanning microscopy (CLSM), FCM, and CCK8 assays. Fluorescence images of Huh1 and Hepa1-6 cells, as shown in Fig. 3A, reveal that the blue fluorescence corresponds to DAPI-stained cell nuclei, while the red fluorescence indicates Cy5-labeled AI NPs. The images demonstrate a time-dependent increase in the uptake of Cy5-AI NPs, with a clear gradient of uptake over time. Additionally, the NPs predominantly distribute within the cytoplasm, confirming that Cy5-AI NPs were successfully internalized by Hepa1-6 and Huh1 cells. FCM analysis (Fig. 3B) also shows a gradual increase in fluorescence intensity over time, reaching its peak at 6 h, further supporting the time-dependent enhancement of AI NPs uptake by Hepa1-6 and Huh1 cells.

Subsequently, as illustrated in Fig. 3C, the cytotoxic effects of AI NPs were assessed using CCK8 assays to evaluate cell viability, Annexin V/PI staining combined with FCM to detect apoptosis, and Calcein-AM/PI staining followed by CLSM imaging. CLSM images of Calcein-AM/PI staining revealed that (Fig. 3D), compared to the Blank group, ART and ICA treatments induced partial tumor cell death, but most cells remained viable. In contrast, ART + ICA and AI NPs treatments resulted in more extensive cell death, with the AI NPs group exhibiting the highest number of dead cells. This indicates that ART and ICA exert satisfactory additive cytotoxic effects in vitro, and this synergistic effect is further enhanced and amplified when encapsulated in AI NPs. Additionally, the CCK8 assay (Fig. 3E) demonstrated a dose-dependent decrease in cell viability for Hepa1-6 and Huh1 cells as the drug concentration increased. This indicates a consistent cytotoxicity trend across all experimental groups, with the relative cytotoxicity following the order: ART or ICA < ART + ICA < AI NPs.

Furthermore, FCM analysis of Annexin V/PI staining (Fig. 3F and G) revealed that compared to the Blank group, ART or ICA treatment increased the apoptosis rate of Hepa1-6 and Huh1 cells. However, AI NPs treatment induced significantly higher apoptosis rates than ART + ICA or other groups, confirming the prominent synergistic cytotoxic effects of ART and ICA when codelivered in the AI NPs nanocarrier. The results in Fig. S7 demonstrated that the protein level of Cleaved-caspase 3 in Hepa1-6 cells markedly increased after treatment with AI NPs, indicating that AI NPs can effectively induce cell apoptosis. These results collectively highlight the enhanced antitumor efficacy of AI NPs, showcasing a promising approach for efficient tumor cell cytotoxicity via combinatorial drug delivery in nanoplatforms.

### AI NPs induce multimodal energy depletion of tumor cells

**Fig. 4.**
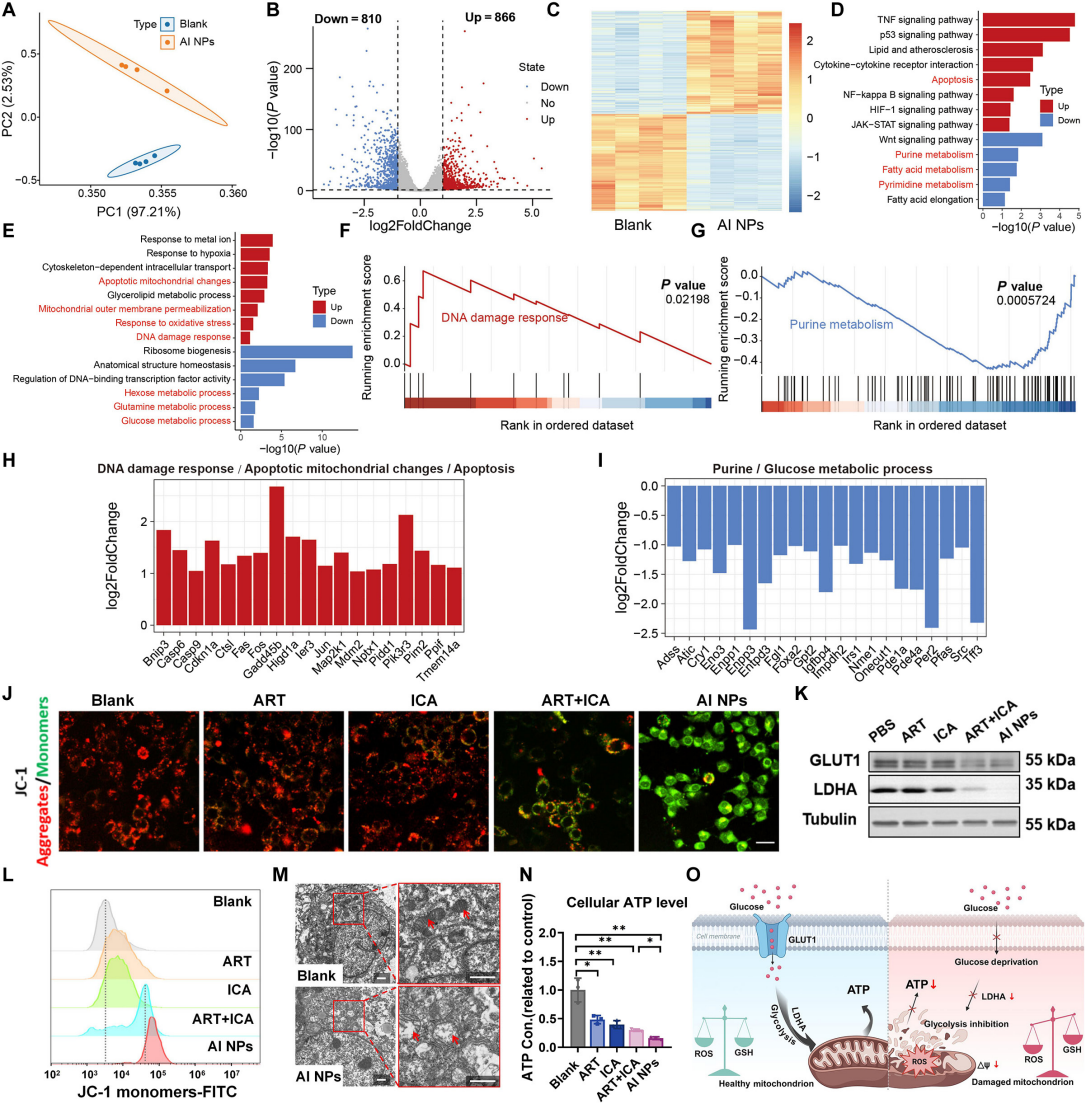
Investigation of the in vitro antitumor mechanisms of AI NPs. (A) Principal component analysis (PCA) of transcriptomic profiles of Hepa1-6 cells treated with PBS or AI NPs. (B) Volcano plot of differentially expressed genes (DEGs) in Hepa1-6 cells treated with PBS or AI NPs. The numbers of down-regulated/up-regulated genes are indicated above. (C) Heatmap showing the relative expression levels of DEGs across samples. (D) KEGG pathways and (E) biological processes enriched by DEGs. (F) GSEA identifying up-regulated DNA damage response pathways and (G) down-regulated purine metabolism pathways. (H) Fold changes in the expression of genes involved in key up-regulated pathways and (I) down-regulated pathways. (J) Fluorescence images of JC-1 staining in Hepa1-6 cells treated with different formulations. Scale bar: 25 μm. (K) Expression levels of glycolysis-related proteins in Hepa1-6 cells treated with different formulations. (L) Flow cytometry analysis of JC-1 staining in Hepa1-6 cells treated with different formulations. (M) Transmission electron microscopy (TEM) images showing mitochondrial damage in Hepa1-6 cells treated with AI NPs. Scale bars: 1 μm. (N) Intracellular ATP levels in Hepa1-6 cells treated with different formulations. Data are presented as mean ± SD (*n* = 3). *P < 0.05; **P < 0.01; one-way ANOVA. (O) Schematic diagram illustrating the in vitro antitumor mechanisms of AI NPs.

To further investigate the antitumor mechanism of AI NPs, a comprehensive analysis was conducted using the RNA sequencing method, mitochondrial damage assessment, and the key indicators analysis of cellular energy metabolism. Differential analysis in the RNA sequencing method provided insights into the tumoricidal effects induced by AI NPs. First, RNA was extracted from Hepa1-6 cells treated with either the Blank (*n* = 4) or AI NPs (*n* = 4) and subjected to transcriptomic sequencing. PCA revealed significant differences in the expression patterns between the 2 groups (Fig. 4A). Volcano plot analysis identified 1,676 DEGs, comprising 810 down-regulated and 866 up-regulated genes (Fig. 4B). Heatmap analysis indicated that these DEGs were evenly distributed across the 2 sample groups (Fig. 4C). Pathway enrichment analysis revealed that the up-regulated genes were primarily involved in mitochondrial apoptosis, oxidative stress, and DNA damage responses, while the down-regulated genes were predominantly associated with metabolic pathways, including glutamine metabolism, glucose metabolism, and purine metabolism (Fig. 4D and E). GSEA of the transcriptomic data further indicated a significant enhancement in DNA damage response posttreatment, with genes associated with DNA damage and apoptosis showing up-regulated expression. Conversely, purine metabolism was significantly reduced, and genes related to energy metabolism were generally down-regulated (Fig. 4F and G). Comparative analysis of apoptosis and autophagy-related markers showed that, in the DNA damage response and mitochondrial changes associated with apoptosis, the ART treatment group exhibited significant up-regulation of these genes, highlighting the substantial impact of ART on the expression of genes involved in DNA damage response, mitochondrial apoptosis, and the induction of cell death (Fig. 4H).

Moreover, in the expression analysis of genes involved in purine/glucose metabolism, the AI NPs treatment group displayed significant down-regulation of multiple genes, suggesting that AI NPs inhibited genes related to purine/glucose metabolism, thereby disrupting cellular energy metabolism (Fig. 4I). These findings collectively demonstrate that AI NPs significantly impair the energy supply system of tumor cells, reducing their intake of essential energy sources and inducing a form of starvation-induced cell death. The combinatorial modulation of energy metabolism by ART and ICA in the AI NPs composite nanomedicine further amplifies its tumoricidal effect.

To further confirm the antitumor mechanism of AI NPs, the effects on mitochondrial damage, key glycolytic protein secretion, mitochondrial morphological changes, and ATP secretion were analyzed. Fluorescence imaging revealed that after ART treatment, cells exhibited strong red JC-1 fluorescence with minimal green fluorescence (Fig. 4J). In contrast, ICA-treated cells showed prominent red and the stronger green fluorescence, indicating the apparent presence of mitochondrial membrane depolarization. Compared to single-agent treatments, ART + ICA cotreatment induced noticeable green fluorescence, suggesting significant changes in mitochondrial membrane potential due to the combined action of both agents. The most pronounced effect was observed in the AI NP-treated cells, where almost no red fluorescence from JC-1 aggregation was detected, and strong green fluorescence was observed, indicating that AI NPs significantly lower mitochondrial membrane potential, thereby promoting apoptosis (Fig. 4J). Then, Western blot analysis (Fig. 4K and Fig. S8A and B) showed that single-agent treatments had a limited impact on glycolysis, whereas ART + ICA and AI NPs markedly decreased the expression of GLUT1 and LDHA, suggesting that the combination treatments effectively suppressed glycolysis and impaired cellular energy supply. Figure S8C quantitatively illustrated intracellular lactate production—an indicator of glycolytic flux—after 24 h treatment. Relative to the blank control, ART alone yielded a modest 12.9% reduction, and ICA produced a comparable decrease. Coadministration of free ART + ICA lowered lactate by 45.0%, confirming additive interference with glycolysis. Strikingly, the AI nanoplatform attenuated lactate to a 90.4% decrease relative to the control group. These data substantiate that the combined nano-formulation potently disrupts the Warburg phenotype, reinforcing the multimodal energy-depletion mechanism proposed. FCM analysis (Fig. 4L) further confirmed these findings, showing the highest fluorescence intensity of JC-1 monomers in the AI NPs group. This result, consistent with the immunofluorescence imaging, indicates a significant change in mitochondrial membrane permeability, leading to a drastic decrease in membrane potential and supporting the conclusion that AI NPs treatment induces substantial apoptosis. Furthermore, the data from TEM (Fig. 4M) revealed healthy mitochondria in untreated cells, characterized by intact outer membranes and dense cristae. In contrast, AI NPs treatment resulted in mitochondrial matrix thinning, decreased electron density, and vacuolization, with cristae shortening and fragmentation due to inner membrane stretching. These observations indicate that AI NPs disrupt mitochondrial structural integrity, impairing normal cellular physiological functions and potentially triggering apoptosis or necrosis. The intracellular ATP content measurement (Fig. 4N) showed a decline in ATP levels in single-agent treated cells compared to the untreated control, likely due to the individual effects of ART and ICA on glycolysis and mitochondrial function. After ART + ICA treatment, ATP levels further decreased, suggesting that the additive effect of both agents exacerbates energy depletion. AI NPs treatment resulted in the lowest ATP levels, likely due to the enhanced cellular uptake of AI NPs compared to the more hydrophobic single agents. Collectively, these data, supported by RNA sequencing and traditional molecular biology assays, as illustrated in Fig. 4O, demonstrate that the multimodal energy depletion strategy employed by AI NPs significantly disrupts mitochondrial function and tumor cell energy metabolism. This comprehensive disruption impairs glucose uptake, blocks glycolysis, depletes ATP levels, and increases oxidative stress, all of which work together to induce tumor cell apoptosis and promote effective tumor cell death.

### AI NPs effectively inhibit Hepa1-6 tumor growth in vivo

**Fig. 5.**
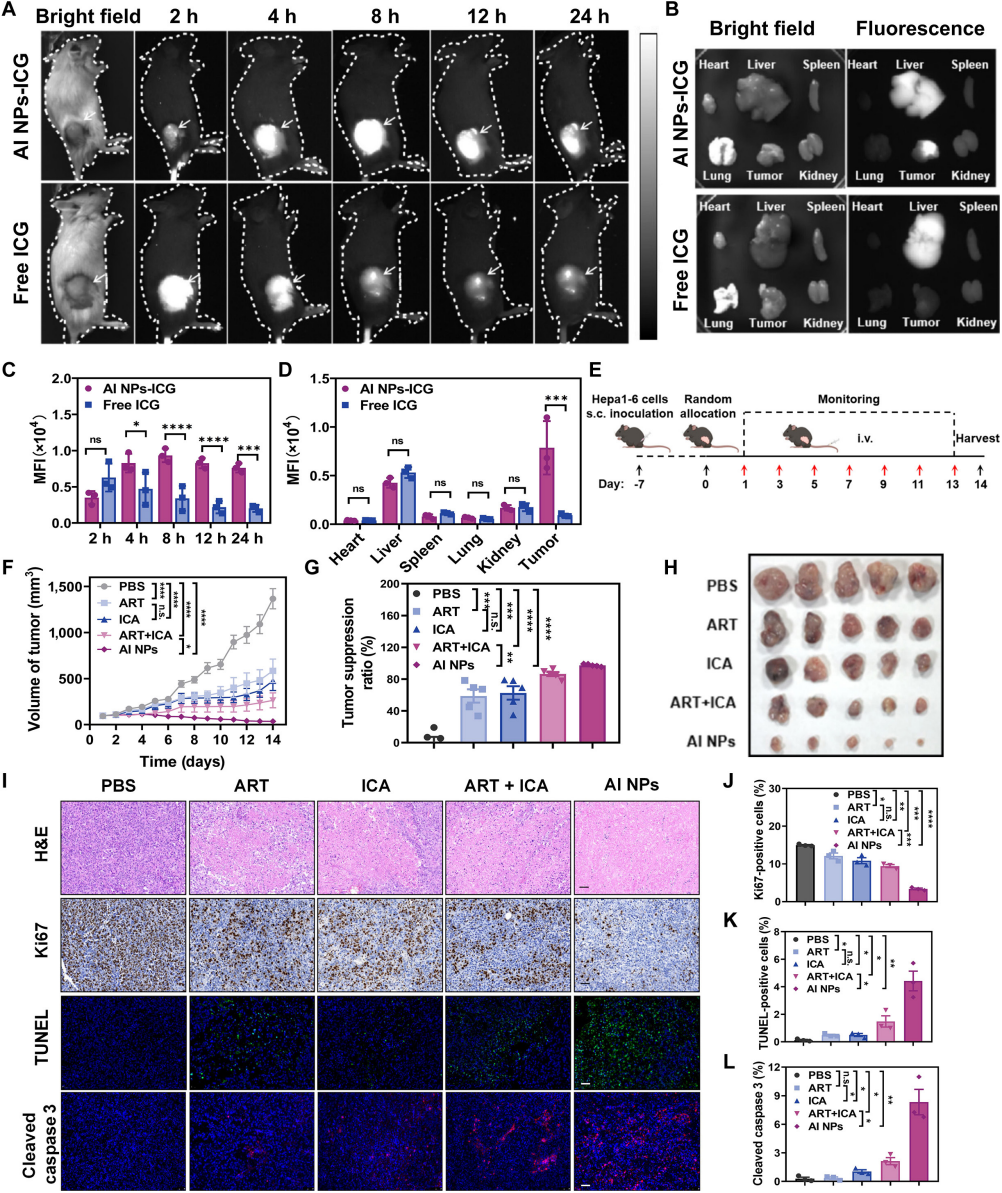
In vivo antitumor efficacy of AI NPs. (A) In vivo imaging and biodistribution of AI NPs-ICG in Hepa1-6 tumor-bearing mice. (B) Ex vivo fluorescence distribution in tissues 24 h after administration of AI NPs-ICG. (C) Quantitative analysis of in vivo fluorescence imaging of AI NPs-ICG. Statistical analysis was conducted with Student’s *t* test. (D) Quantitative analysis of ex vivo fluorescence in tissues 24 h after administration of AI NPs-ICG. Statistical analysis was conducted with Student’s *t* test. (E) Schematic diagram of the treatment protocol for Hepa1-6 tumor-bearing mice. (F) Tumor growth curves in mice treated with different therapeutic groups. Two-way ANOVA. (G) Tumor inhibition rates after treatment with different therapeutic groups. One-way ANOVA. (H) Tumor images of mice after treatment with different therapeutic groups. (I) Histological analysis of tumors after treatment, including H&E staining, Ki67 staining, TUNEL staining, and Cleaved-caspase 3 staining. Scale bars: 50 μm. (J) Quantitative analysis of Ki67 staining, (K) TUNEL staining, and (L) Cleaved-caspase 3 staining in tumors after treatment. Data are presented as mean ± SD. *P < 0.05; **P < 0.01; ***P < 0.001; ****P < 0.0001; n.s., not significant. One-way ANOVA.

Building on the demonstrated multimodal ability of AI NPs to regulate tumor cell energy metabolism in vitro, the in vivo antitumor effects were further evaluated. Fluorescence distribution in mice revealed that AI NPs-ICG accumulated at the tumor site within 4 h, with significant retention observed at 24 h, whereas free ICG fluorescence was nearly absent at 24 h. This suggests that ICG-loaded AI NPs exhibit excellent tumor targeting and prolonged retention at the tumor site (Fig. 5A). Semiquantitative analysis indicated that the fluorescence intensity of AI NPs-ICG increased over time, peaking at approximately 8 h before gradually decreasing, whereas free ICG showed the strongest fluorescence at around 2 h postinjection, followed by a decrease over time (Fig. 5C). This indicates that AI NPs-ICG likely have a longer half-life in the bloodstream, enabling sustained delivery to tumor sites, with fluorescence intensity gradually increasing to its peak. Then, the ex vivo organ imaging further confirmed that the fluorescence in the tumor was significantly higher than in other organs, indicating that AI NPs-ICG effectively accumulated at the tumor site, thereby enhancing the potential for therapeutic efficacy (Fig. 5B). In tumor tissues, the mean fluorescence intensity (MFI) of AI NPs-ICG was significantly higher than that of free ICG, reflecting greater accumulation at the tumor site, while no significant difference was observed in normal tissues such as the heart, liver, spleen, lungs, and kidneys (Fig. 5D). In the in vivo antitumor study, mice were subcutaneously implanted with Hepa1-6 cells, and then randomly divided into different treatment groups. From day 1, intravenous injections were administered every 2 days, with monitoring of mouse conditions until day 14, when samples were collected or mice were euthanized (Fig. 5E). In addition, no statistically significant differences were observed in complete blood count analysis and blood biochemical indices between the treatment group and the PBS group (Figs. S9 and S10), further confirming that the dosage used in this study had low systemic toxicity and a good safety profile. To further validate safety, H&E staining of major organs (heart, liver, spleen, lung, and kidney) was conducted, with results showing no observable tissue damage or inflammatory responses (as shown in Fig. S11). Collectively, these systematic safety assessment experiments provide robust evidence for the reliable biosafety profile of AI NPs. Tumor volume analysis on day 14 showed that tumors in the PBS-treated group had grown to approximately 1,500 mm^3^, demonstrating the high invasiveness of Hepa1-6 cells (Fig. 5F). The body weight of mice in all groups exhibited similar stable trend with different treatments (Fig. S12A), indicating that AI NPs exhibit good biocompatibility. Notably, compared to the PBS group, all treatment groups exhibited partial tumor growth inhibition, while the AI NPs group showed the most significant antitumor effect, achieving a tumor inhibition rate of 97.34%, nearly completely suppressing tumor growth (Fig. S12B and C). The inhibition rates for the free ART, ICA, and ART + ICA groups were 58.76%, 62.61%, and 86.35%, respectively, highlighting the superior antitumor efficacy of AI NPs (Fig. 5G). The tumor morphology images and tumor weight statistics were consistent with the aforementioned results, demonstrating that AI NPs exhibited the most significant tumor inhibition rate (Fig. 5H and Fig. S12C). In addition, the histological staining images indicated that AI NPs treatment led to a more pronounced induction of tumor cell apoptosis compared to the PBS group, confirming the apoptotic potential of AI NPs (Fig. 5I). The proliferation rate, as measured by Ki67 staining, was reduced in all treatment groups compared to PBS, with AI NPs exhibiting the most significant inhibition of cell proliferation (Fig. 5J). Additionally, the percentage of TUNEL-positive cells was higher in the ART, ICA, ART + ICA, and AI NPs groups, with the highest percentage observed in the AI NP-treated group, further supporting its ability to induce apoptosis (Fig. 5K). Cleaved-caspase 3 staining revealed the highest levels of Cleaved-caspase 3 in tumors treated with AI NPs, further confirming its potent antitumor effects (Fig. 5L).

### AI NPs effectively inhibit different tumor growth in vivo

**Fig. 6.**
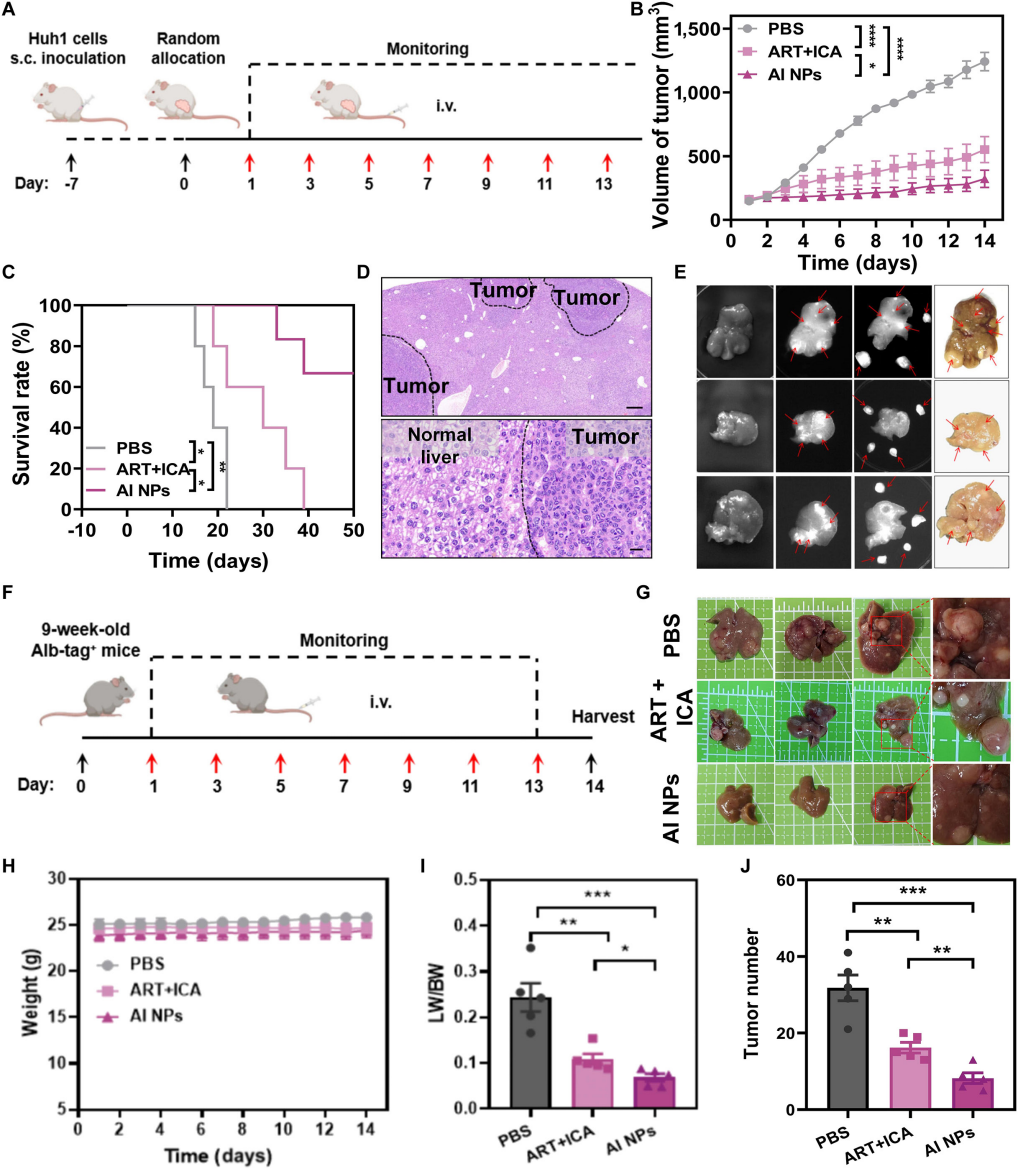
The therapeutic effects of AI NPs in different tumor models. (A) Schematic diagram of the treatment protocol for Huh1 tumor-bearing mice. (B) Tumor growth curves in Huh1 tumor-bearing mice treated with different therapeutic groups. Two-way ANOVA. (C) Survival rates of Huh1 tumor-bearing mice after treatment with different therapeutic groups. The difference between 2 groups was assessed by Kaplan–Meier survival analysis (log-rank test). (D) H&E staining of liver tissues in Alb-tag^+^ mice. (E) Biodistribution of AI NPs-ICG in the livers of Alb-tag^+^ mice. (F) Schematic diagram of the treatment protocol for Alb-tag^+^ mice. (G) Images of liver tumor nodules in Alb-tag^+^ mice after treatment with different therapeutic groups. (H) Body weight monitoring of Alb-tag^+^ mice during treatment with different therapeutic groups. One-way ANOVA. (I) Liver-to-body weight ratios and (J) tumor number in Alb-tag^+^ mice after treatment with different therapeutic groups. Data are presented as mean ± SD (*n* = 5). *P < 0.05; **P < 0.01; ***P < 0.001. One-way ANOVA.

In additional experiments using a human liver cancer cell line (Huh1), the therapeutic efficacy of AI NPs was similarly evaluated. After random grouping, mice were treated with different drug formulations and monitored for tumor growth from day 1 to day 14 (as illustrated in Fig. 6A). The body weight of mice was continuously monitored during the tumor treatment period (Fig. S13A), confirming the biosafety of AI NPs. Huh1 tumor volume analysis over time showed significantly slower tumor growth in the treatment groups compared to the PBS group, with the AI NPs group exhibiting the most substantial reduction in tumor growth (Fig. 6B and Fig. S13B), consistent with the previous results in Hepa1-6 tumor models. Survival analysis indicated that the PBS-treated group had a rapid decline in survival, whereas the ART + ICA and AI NPs groups showed slower survival rate declines, with the AI NPs group maintaining the highest survival rate over a longer period, demonstrating the prolonged survival-promoting effect of AI NPs (Fig. 6C).

Finally, HCC transgenic mice (Alb-tag^+^ mice) were used to investigate the therapeutic efficacy of AI NPs. At 11 weeks of age, Alb-Tag^+^ mice were highly expressed in microscopic hypercellular nodules observed within these livers (Fig. 6D). Hence, approximately 11-week-old Alb-Tag^+^ mice were selected for ex vivo imaging to detect the distribution of AI NPs in the liver. The results of Fig. 6E revealed that the fluorescence intensity of AI NPs-ICG in liver tumor nodules was significantly higher than that in normal liver tissues, indicating that AI NPs can accumulate in the diseased regions of the liver, providing a basis for further pharmacological studies. The treatment protocol for Alb-tag^+^ mice is illustrated in Fig. 6F. Image analysis of the liver tissue showed that AI NP-treated tumors were significantly smaller compared to the PBS group, indicating effective inhibition of tumor growth (Fig. 6G). During the treatment period, the body weight of mice was also monitored as an indicator. The results showed no significant trend of change in body weight during the treatment (Fig. 6H). Further analysis of liver weight-to-body weight ratios (LW/BW), tumor number, and liver weight revealed that both AI NPs and ART + ICA treatment groups showed significantly lower LW/BW ratios compared to the PBS group, with the AI NPs treatment group showing the most significant inhibition (Fig. 6I and J and Fig. S14) [33,34]. These results demonstrate that AI NPs effectively inhibit tumor growth and promote tumor cell apoptosis. The AI NPs treatment showed superior tumor suppression and prolonged survival in comparison to other treatment groups, highlighting their potential for enhancing antitumor efficacy.

### AI NPs inhibit tumor angiogenesis

**Fig. 7.**
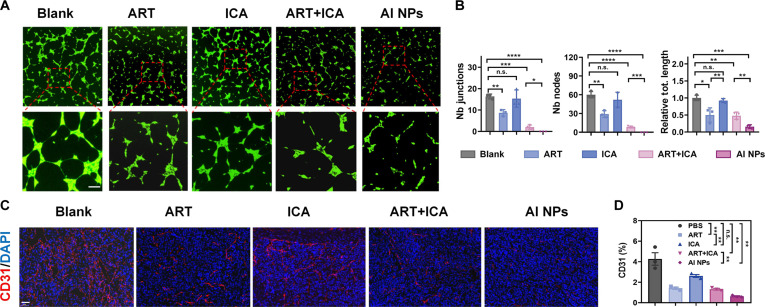
Antiangiogenic effects of AI NPs. (A) Fluorescence images of vascular formation in mouse pancreatic endothelial cells (MS1) treated with different drug formulations. Scale bar: 25 μm. (B) Quantitative analysis of vascular formation in MS1 cells, including the number of intersections, nodes, and total vessel length after treatment with different drug formulations. (C) Fluorescence images of CD31 staining in tumor tissues of Hepa1-6 tumor-bearing mice treated with different drug formulations. Scale bar: 50 μm. (D) Quantitative analysis of CD31 staining in tumor tissues of Hepa1-6 tumor-bearing mice treated with different drug formulations. Data are presented as mean ± SD (*n* = 3). *P < 0.05; **P < 0.01; ***P < 0.001; ****P < 0.0001; n.s., not significant. One-way ANOVA.

Data from Fig. 4 indicate that the robust tumor inhibition observed in the in vivo experiments depicted in Figs. 5 and 6 is closely associated with cellular energy depletion and the regulation of energy metabolism in tumor cells. Furthermore, the results demonstrate that AI NPs significantly inhibit tumor angiogenesis. Fluorescence microscopy images and quantitative analysis revealed that the numbers of Nb junctions, Nb nodes, and relative total length for ART, ICA, ART + ICA, and AI NPs were reduced compared to the Blank group, with the AI NPs showing the most pronounced decrease (Fig. 7A and B). Additionally, examination of tumor vascular distribution showed that AI NPs treatment markedly suppressed angiogenesis, contributing to tumor growth inhibition by limiting energy supply (Fig. 7C and D). These findings suggest that AI NPs disrupt mitochondrial function and energy metabolism, promote starvation-induced apoptosis and cell death, and effectively inhibit tumor vascularization in vivo. The combined effect of these mechanisms provides a reliable foundation for tumor energy metabolism regulation and suppression, contributing to the combination therapeutic effects observed in Fig. 5.

### AI NPs activate tumor immunity

**Fig. 8.**
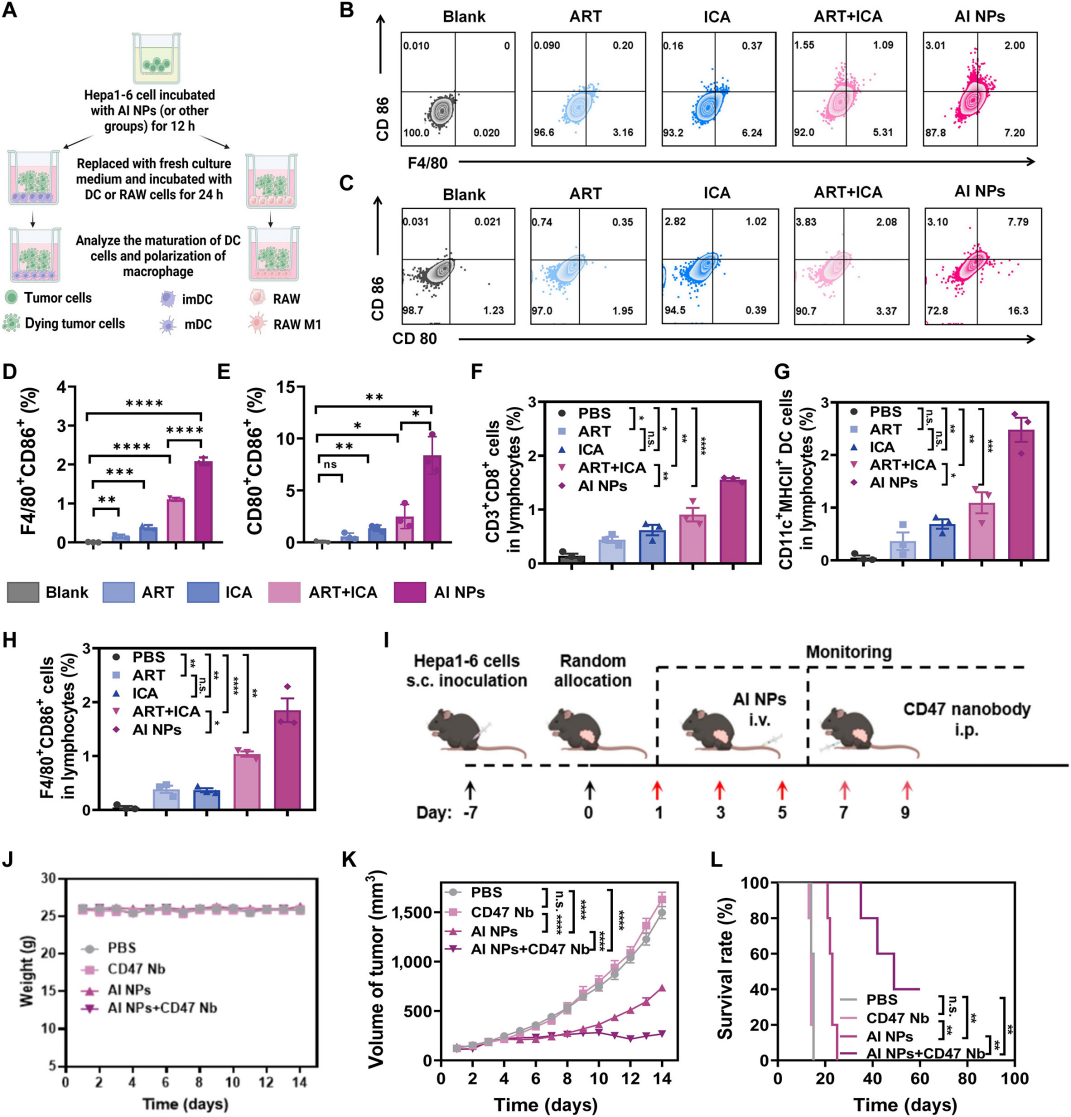
Activation of systemic immunity in mice by AI NPs. (A) Schematic diagram of in vitro immune cell activation studies. (B) Expression levels of macrophage polarization markers induced by supernatants from Hepa1-6 cells treated with different drug formulations for 24 h. (C) Expression levels of dendritic cell (DC) maturation markers induced by supernatants from Hepa1-6 cells treated with different drug formulations for 24 h. (D) The quantitative results of polarized macrophages and (E) mature dendritic cells. One-way ANOVA. (F) The quantitative result of CD3^+^CD8^+^ T cells in tumor tissues of Hepa1-6 tumor-bearing mice treated with different drug formulations. One-way ANOVA. (G) The quantitative result of mature DCs (CD11c^+^MHCII^+^) in tumor tissues of Hepa1-6 tumor-bearing mice treated with different drug formulations. One-way ANOVA. (H) The quantitative result of polarized macrophages (F4/80^+^CD86^+^) in tumor tissues of Hepa1-6 tumor-bearing mice treated with different drug formulations. One-way ANOVA. (I) Schematic diagram of the treatment protocol for Hepa1-6 tumor-bearing mice with combined therapy of AI NPs and CD47 nanobody. (J) Body weight monitoring of Hepa1-6 tumor-bearing mice treated with combined therapy of AI NPs and CD47 nanobody. (K) Tumor growth curves in Hepa1-6 tumor-bearing mice treated with different therapeutic regimens. Two-way ANOVA. (L) Survival rates of Hepa1-6 tumor-bearing mice after treatment with different therapeutic regimens. The difference between 2 groups was assessed by Kaplan–Meier survival analysis (log-rank test);*P < 0.05; **P < 0.01; ***P < 0.001; ****P < 0.0001; n.s., not significant.

Furthermore, the fluorescence staining of CALR expression (Fig. S15) reveals that AI NPs, through their regulation of tumor energy metabolism, more effectively induce CALR production in tumor cells. This finding suggests that AI NPs trigger a starvation-induced immunogenic cell death (ICD) response following energy depletion, which is consistent with previously reported work [35–37]. Consequently, subsequent experiments were systematically designed to assess the immune response associated with AI NPs treatment by evaluating relevant immune markers. As depicted in Fig. 8A, Hepa1-6 tumor cells were initially incubated with AI NPs (or other treatments) for 12 h, followed by replacement with fresh medium and subsequent coculture with DCs or RAW 264.7 macrophages for 24 h. This design aimed to elucidate the impact of AI NPs on DC maturation and macrophage polarization. Flow cytometric analysis demonstrates a pronounced increase in the percentage of F4/80^+^CD86^+^ macrophages in the AI NPs group compared to Blank, ART, ICA, or ART + ICA groups, suggesting that AI NPs skew macrophages more effectively toward the M1 phenotype (Fig. 8B and D). Then, this study also examined the status of DCs. The results showed that tumor cells treated with AI NPs significantly stimulated DC maturation (Fig. 8C and E), indicating that AI NP-treated tumor cells may elicit antitumor immunity. Quantification of these populations further confirms that AI NPs treatment achieves the most significant up-regulation of pro-inflammatory markers among all tested conditions.

Turning to the in vivo context, Hepa1-6 tumor-bearing mice were administered different treatments to assess the activation of systemic immunity. The gating process for the FCM results is illustrated in Fig. S16. Tumor tissues from the AI NPs group displayed a significantly greater infiltration of CD3^+^CD8^+^ T cells (Fig. 8F) and a higher proportion of mature DCs (CD11c^+^MHC II^+^; Fig. 8G) compared to those receiving other formulations. Additionally, macrophages residing in the tumor microenvironment demonstrated a shift toward a pro-inflammatory phenotype (F4/80^+^CD86^+^) under AI NPs treatment (Fig. 8H), underscoring the robust immunostimulatory capacity of this approach.

In summary, these findings highlight that AI NPs effectively shape the tumor immune microenvironment by orchestrating macrophage polarization, driving DC maturation, and promoting cytotoxic T-cell infiltration. Results showed that AI NPs treatment notably induced macrophage polarization, with a marked increase in the M1 macrophage population (Fig. 8H). Together, these data provide compelling evidence for the combinatorial potential of energy-deleted tumor cell death and immune activation mediated by AI NPs, suggesting that AI NPs can block tumor energy supply through multiple pathways to inhibit tumor growth, and remodel the TME to enhance immune activity.

CD47 is a transmembrane protein widely expressed on the surface of both normal and tumor cells. It interacts with signal regulatory protein alpha (SIRPα) on macrophages to deliver a “don’t eat me” signal, thereby inhibiting the phagocytosis of tumor cells by macrophages. Building on the findings regarding tumor-associated macrophage (TAM) infiltration and DC activation, further investigation was conducted to assess whether combining AI NPs with CD47 nanobody could additively inhibit tumor growth and enhance immunotherapy. As shown in Fig. 8I, Hepa1-6 cells were subcutaneously implanted into mice, followed by random grouping and intravenous injections of AI NPs on days 1, 3, and 5, with an intraperitoneal injection of CD47 nanobody on days 7 and 9. Tumor growth was monitored throughout the treatment.

During the treatment period, the body weight of mice was also monitored as an indicator. The results showed no significant trend of change in body weight during the treatment (Fig. 8J). Tumor volume and survival analyses indicated that while the CD47 nanobody alone did not inhibit tumor growth, the AI NPs treatment significantly suppressed tumor growth (Fig. 8K and Fig. S17). However, tumor volume increased upon cessation of AI NPs treatment. Notably, incorporation of CD47 nanobodies into the AI NP formulation further suppressed tumor progression. All tumors in this treatment group remained inhibited throughout the therapeutic period, with persistently slow growth observed even 10 days posttreatment cessation (Fig. 8K and Fig. S17). Survival analysis also confirmed these findings (Fig. 8L). In conclusion, these experimental results demonstrate that AI NPs inhibit tumor growth through multifaceted suppression of energy metabolism while simultaneously reshaping the TME to activate immune responses, particularly through the polarization of M1 macrophages and the maturation of DCs, thereby enhancing tumor immunotherapy. Moreover, the combination of AI NPs with CD47 nanobodies further enhances tumor suppression and improves mouse survival rates.

## Conclusion

In conclusion, this study presents a novel multimodal energy depletion strategy for malignant tumor therapy, utilizing an ART/ICA hybrid nanoplatform. By simultaneously targeting angiogenesis, glucose uptake, and mitochondrial function, this approach successfully addresses the limitations of single-pathway interventions. The GSH-responsive disulfide linkages in the nanomedicine facilitated controlled, tumor-selective drug release, enhancing therapeutic stability and bioavailability. Comprehensive in vitro and in vivo evaluations demonstrated that this platform effectively disrupted tumor energy metabolism, induced apoptosis, and significantly improved tumor inhibition rates, achieving over 97% in subcutaneous tumor models—far surpassing ART or ICA alone. Furthermore, FCM analyses confirmed a reshaped tumor microenvironment, characterized by increased cytotoxic CD8^+^ T-cell infiltration and DC maturation. The addition of a CD47-targeting nanobody further amplified immune activation, contributing to a robust and sustained antitumor response. These findings underscore the potential of multimodal metabolic disruption as a transformative therapeutic strategy for malignant tumor, offering a promising path to improving treatment efficacy and long-term outcomes.

## Ethical Approval

The research was approved by the Institutional Animal Ethics Committee of Shenzhen People’s Hospital (Approval No. AUP-250423-LZJ-0456-1).

## Data Availability

All data relevant to the study are included in the article or uploaded as supplementary information.
