# Supplementary material for: A Multimodal Energy-Depletion Strategy for Cooperative Tumor Metabolism Regulation in Enhanced Cancer Therapy
Source: Biomater Res. 2025 Nov 17;29:0246. doi: 10.34133/bmr.0246 (PMC12620624; doi:10.34133/bmr.0246)
Supplement: Supplementary 1 — Figs. S1 to S17 [file bmr.0246.f1.docx]

# Supporting Information

**A Multi-Modal Energy-Depletion Strategy for Cooperative Tumor Metabolism Regulation in Enhanced Cancer Therapy**

*Jingbo Ma ^1, #^, Kun Chen ^2, #^, Xiaoyong Zhang ^3, #^, Yanni Lou ^4, #^, Yunmeng Bai ^5^, Yinkwan Wong ^5^, Lei Zheng ^7^, Longying Li ^2^,YanWei Hu ^8 *^, Zhijie Li ^5, *^, Feng Qiu ^1, *^, Jigang Wang ^1, 2, 5, 6, 7*^*

^1^ School of Chinese Materia Medica, Tianjin University of Traditional Chinese Medicine, Tianjin 301617, P. R. China.

^2^ Center for Drug Research and Development, Guangdong Provincial Key Laboratory for Research and Evaluation of Pharmaceutical Preparations, Guangdong Pharmaceutical University, Guangzhou, 510006, P. R. China.

^3^ Guangdong Provincial Key Laboratory for Prevention and Control of Major Liver Diseases; Hepatology Unit and Department of Infectious Diseases, Nanfang Hospital, Southern Medical University, Guangzhou 510515, P. R. China.

^4^ Chief Physician, Department of Integrative Oncology, China-Japan Friendship Hospital, Beijing 100029, China.

^5^ Department of Nephrology, Shenzhen Key Laboratory of Kidney Diseases, Guangdong Provincial Clinical Research Center for Geriatrics, Shenzhen Clinical Research Centre for Geriatrics, Shenzhen People’s Hospital (The Second Clinical Medical College, Jinan University; The First Affiliated Hospital, Southern University of Science and Technology), Shenzhen 518020, China.

^6^ State Key Laboratory for Quality Ensurance and Sustainable Use of Dao-di Herbs, Artemisinin Research Center, and Institute of Chinese Materia Medica, China Academy of Chinese Medical Sciences, Beijing 100700, China..

^7^ Guangdong Provincial Key Laboratory of New Drug Screening, School of Pharmaceutical Sciences, Southern Medical University, Guangzhou 510515, P. R. China.

^8^ Department of Laboratory Medicine, Beijing Chao-Yang Hospital, Capital Medical University, Beijing, China.

^*^Corresponding authors:

Jigang Wang, E-mail addresses: [jgwang@icmm.ac.cn](mailto:wangjigang@u.nus.edu;).

Feng Qiu, E-mail addresses: [fengqiu20070118@163.com](mailto:fengqiu20070118@163.com;).

Zhijie Li, E-mail addresses: [li.zhijie@szhospital.com](mailto:li.zhijie@szhospital.com).

Yanwei Hu, E-mail addresses: ywhu@mail.ccmu.edu.cn.

^#^ Jingbo Ma, Kun Chen, Xiaoyong Zhang and Yanni Lou contributed equally to the work.


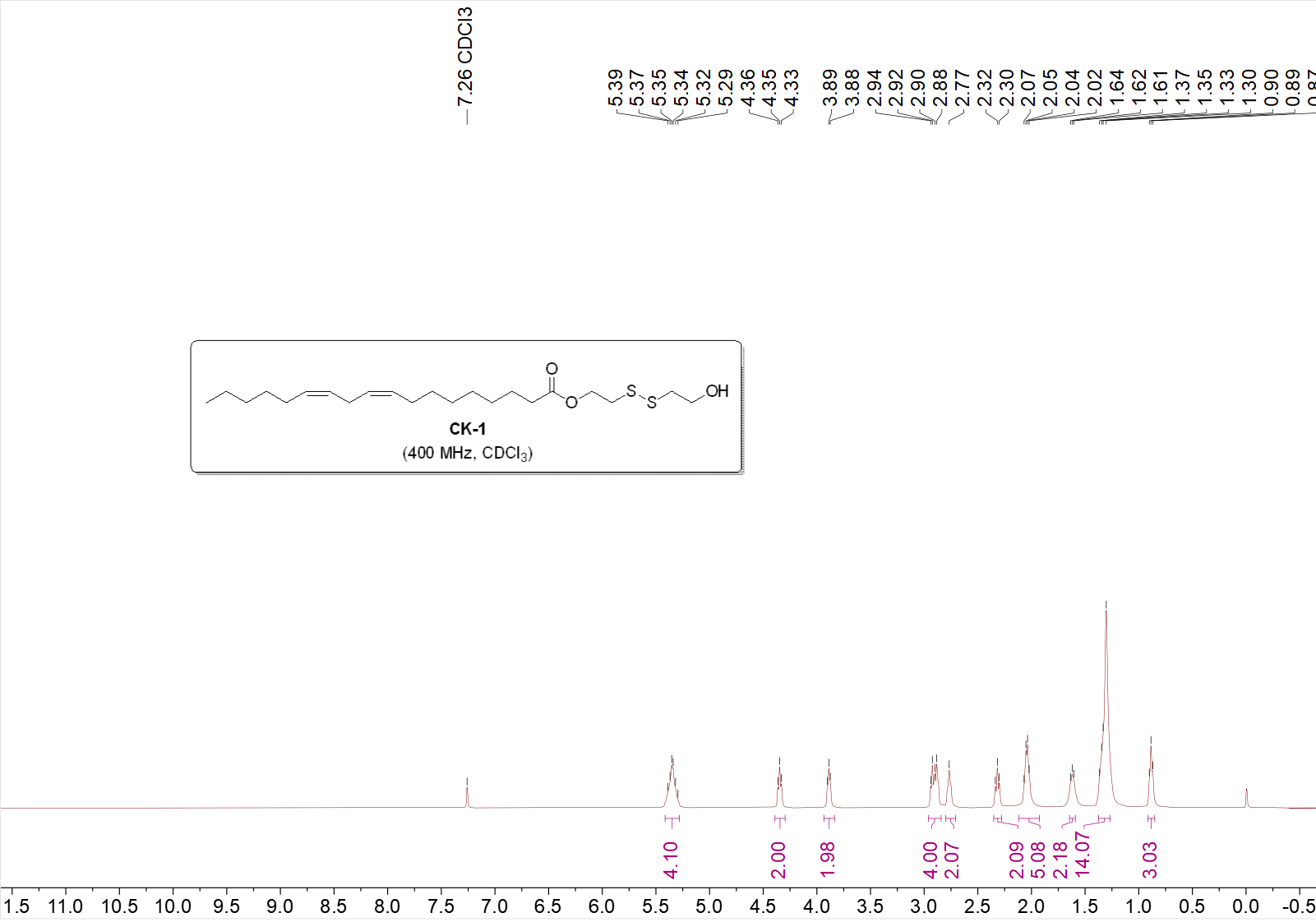


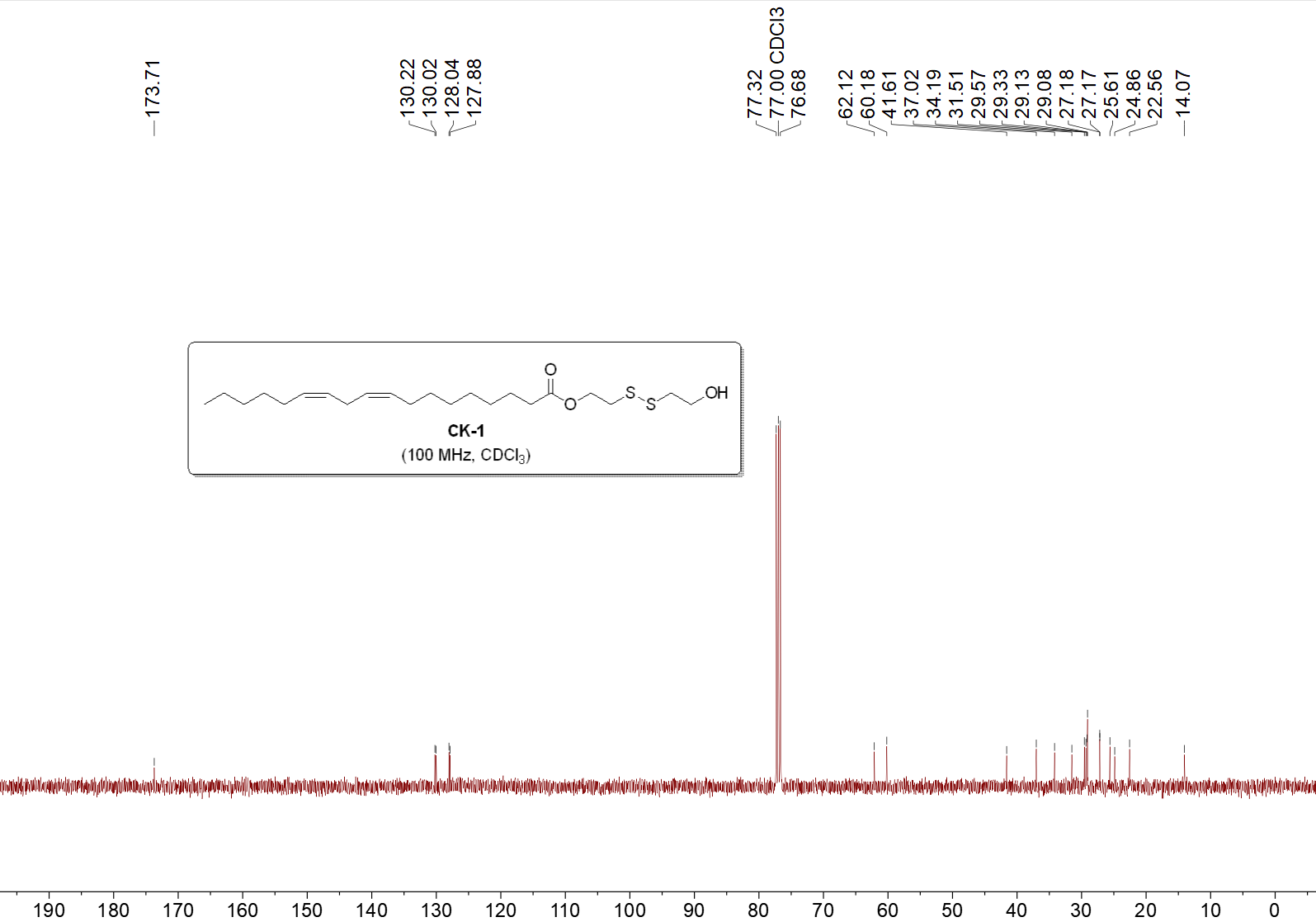


**Fig. S1.** ^1^H NMR (400 MHz, CDCl_3_, ppm) and ^13^C NMR (100 MHz, CDCl_3_, ppm) spectras of CK-1.


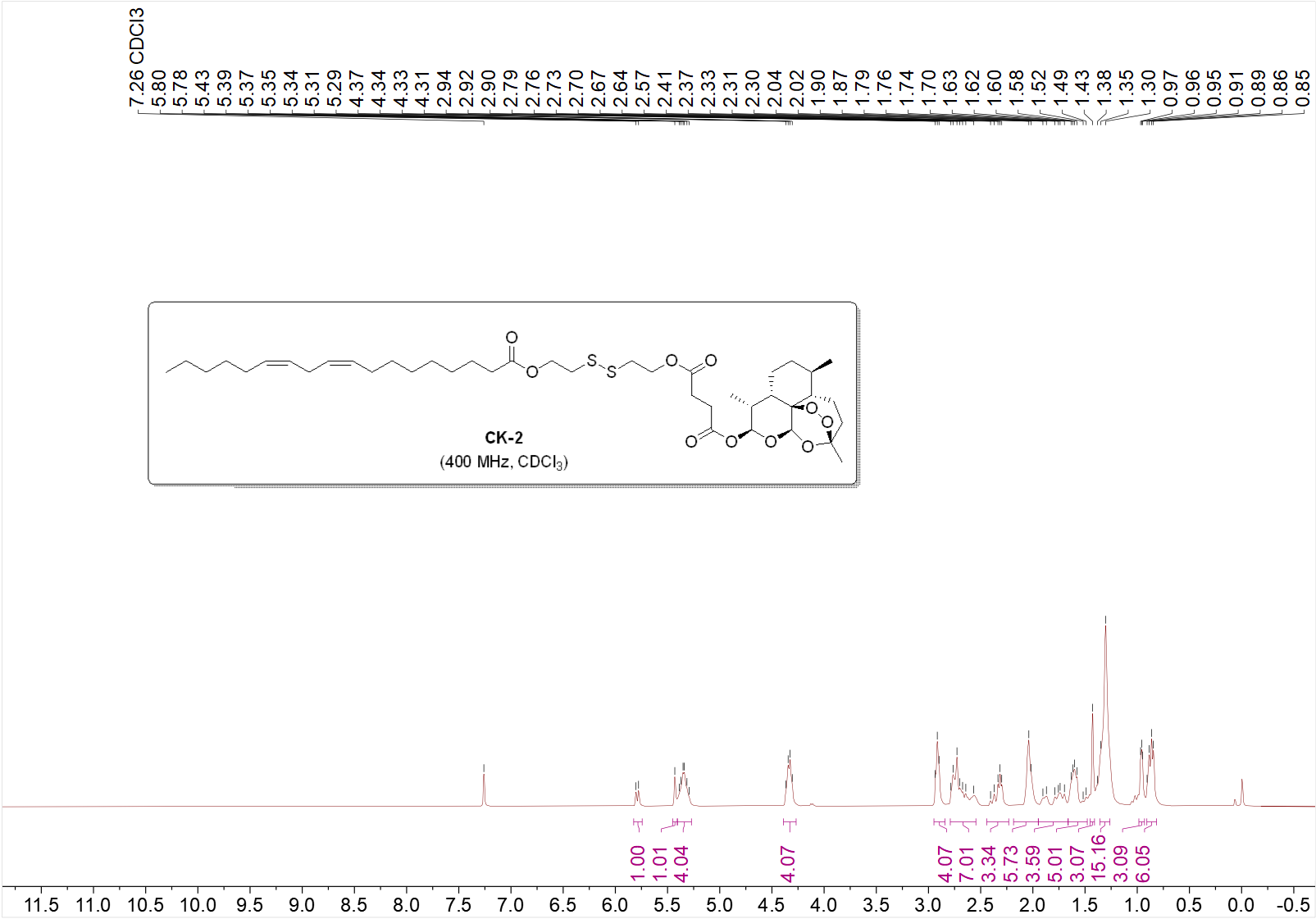


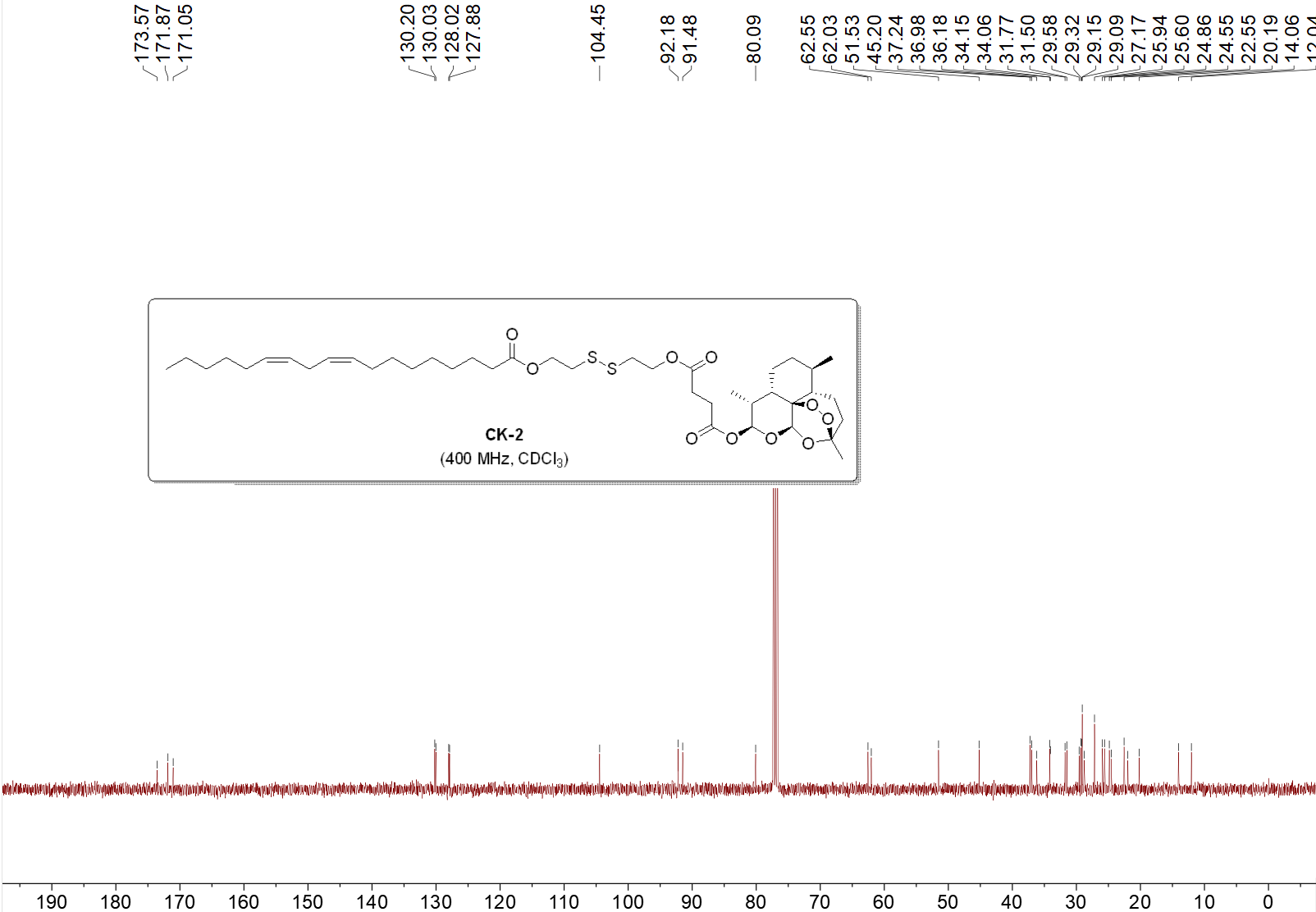


**Fig. S2.** ^1^H NMR (400 MHz, CDCl_3_, ppm) and ^13^C NMR (100 MHz, CDCl_3_, ppm) spectras of CK-2 (Pre-ART).


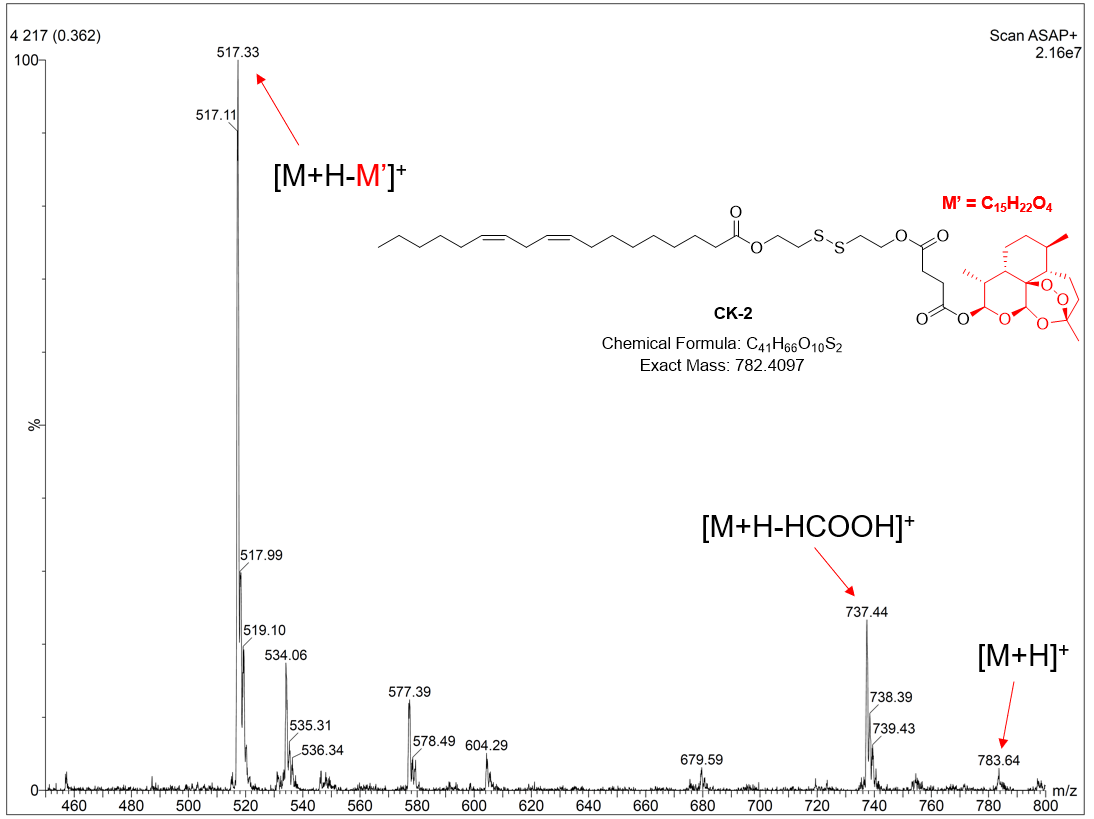


**Fig. S3.** MS (ESI) spectra of CK-2 (Pre-ART).

| **CHROMATOGRAPHYREPORT** | |
| --- | --- |
|  | |
| Column | Diamonsil C18 |
| Column Size | 5 μm, 250 x 4.6 mm |
| Injection | 10 uL |
| Mobile phase | CH_3_CN: H_2_O = 90: 10 |
| Flow rate | 1 mL/min |
| Wavelength | UV 200 nm |
| Temperature | 30 ^o^C |
| Sample solution | 1.5 mg/mL in CH_3_CN |


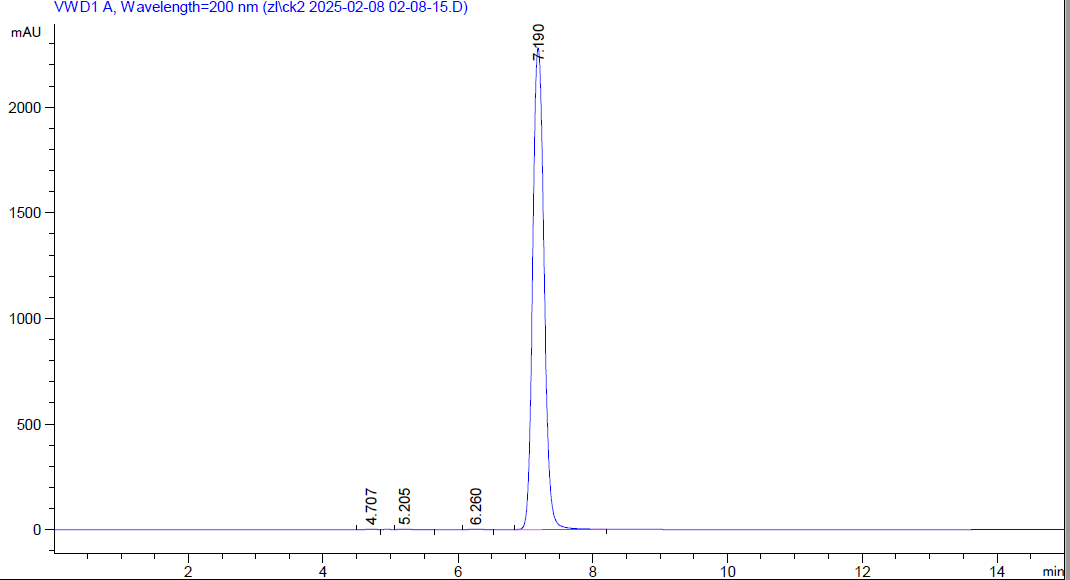


| **Peak** | **RT (min)** | **Height (mAU)** | **Area (mAU*s)** | **Area %** |
| --- | --- | --- | --- | --- |
| 1 | 4.707 | 2.07176 | 15.58699 | 0.0585 |
| 2 | 5.205 | 2.60666 | 23.77635 | 0.0893 |
| 3 | 6.260 | 2.56014 | 23.27803 | 0.0874 |
| 4 | 7.190 | 2279.46313 | 2.65718e4 | 99.7648 |

**Fig. S4.** HPLC spectra of CK2 (Pre-ART).


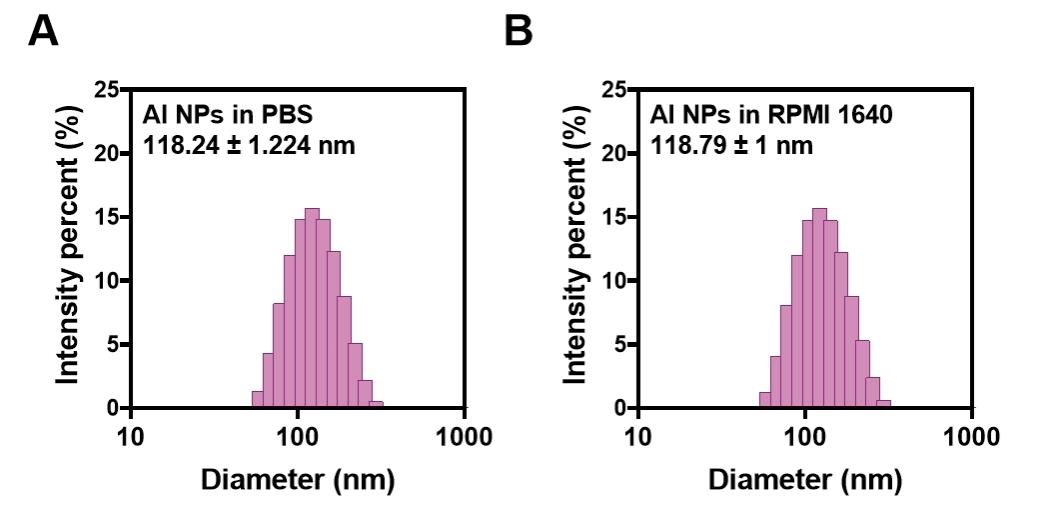


**Fig. S5.** Size distribution of AI NPs self-assembly in (A) PBS and (B) RPMI 1640 culture medium. Data are presented as mean ± S.D. (n = 3).


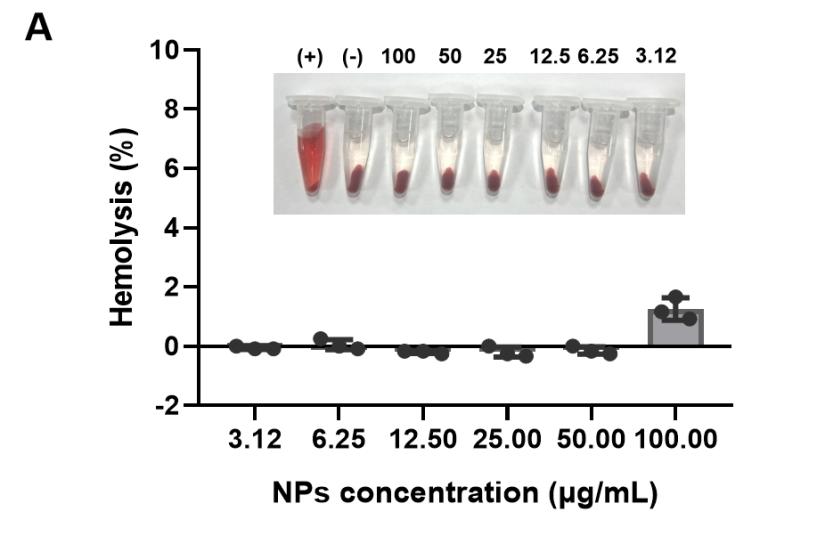


**Fig. S6.** Hemolysis of red blood cells under different concentrations of AI NPs. Data are presented as mean ± S.D. (n = 3).


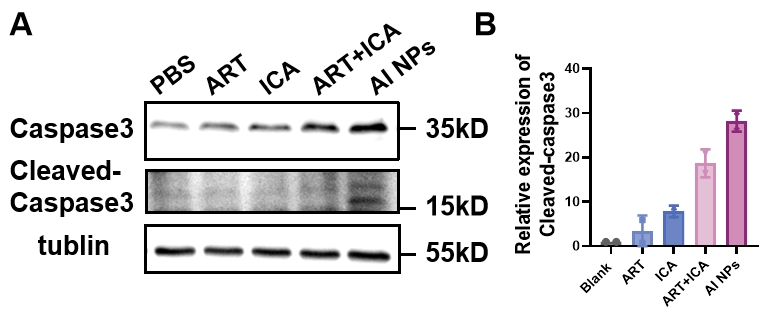


**Fig. S7.** (A) Expression levels of Caspase 3 and Cleaved-Caspase 3 in Hepa1-6 cells after apoptosis induction by different drug treatments. (B) Western blot quantification of Cleaved-caspase3 protein expression levels in Hepa1-6 cells after apoptosis induction by different drug treatments.


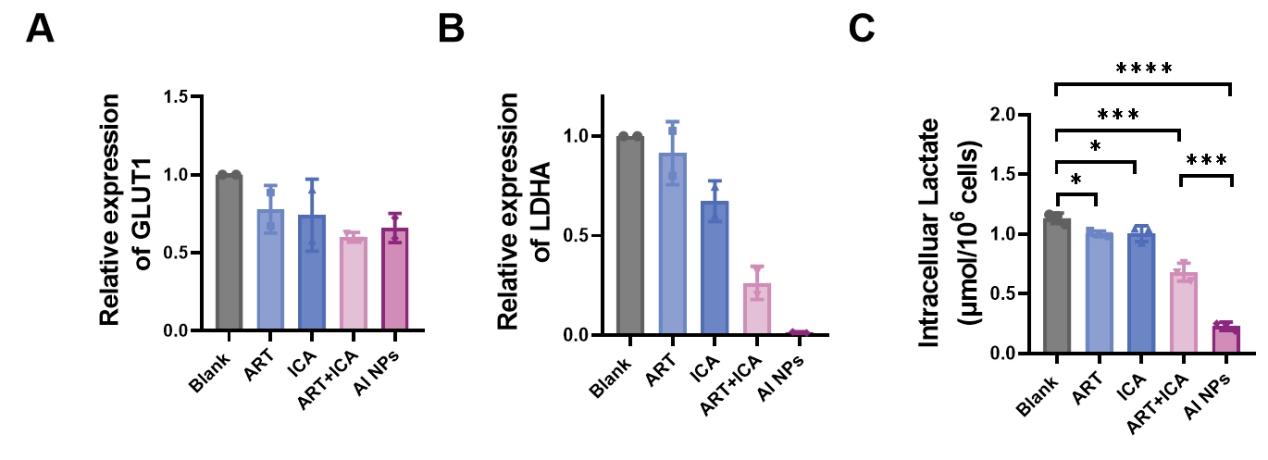


**Fig. S8.** Western blot quantification of (A) GLUT1 and (B) LDHA protein expression levels in Hepa1-6 cells after apoptosis induction by different drug treatments. (C) Intracellular lactate levels in Hepa1-6 cells treated with different drug. Data are presented as mean ± S.D. (n = 3). *p<0.05; **p<0.01; ***p<0.001; ****p<0.0001. One-way Anova.


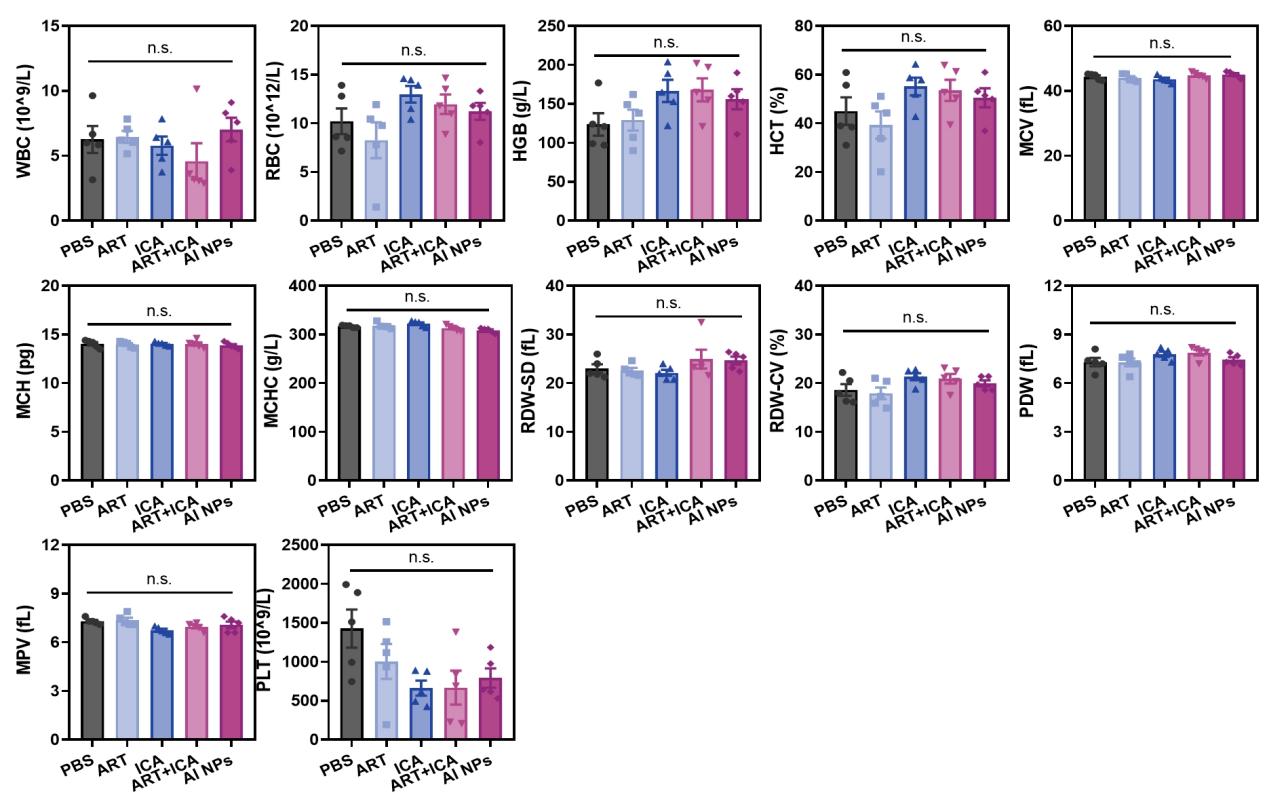


**Fig. S9.** Routine blood tests of Hepa1-6 tumor-bearing mice after treatment with different therapeutic groups. Data are presented as mean ± S.D. (n = 5). n.s., not significant. One-way Anova.


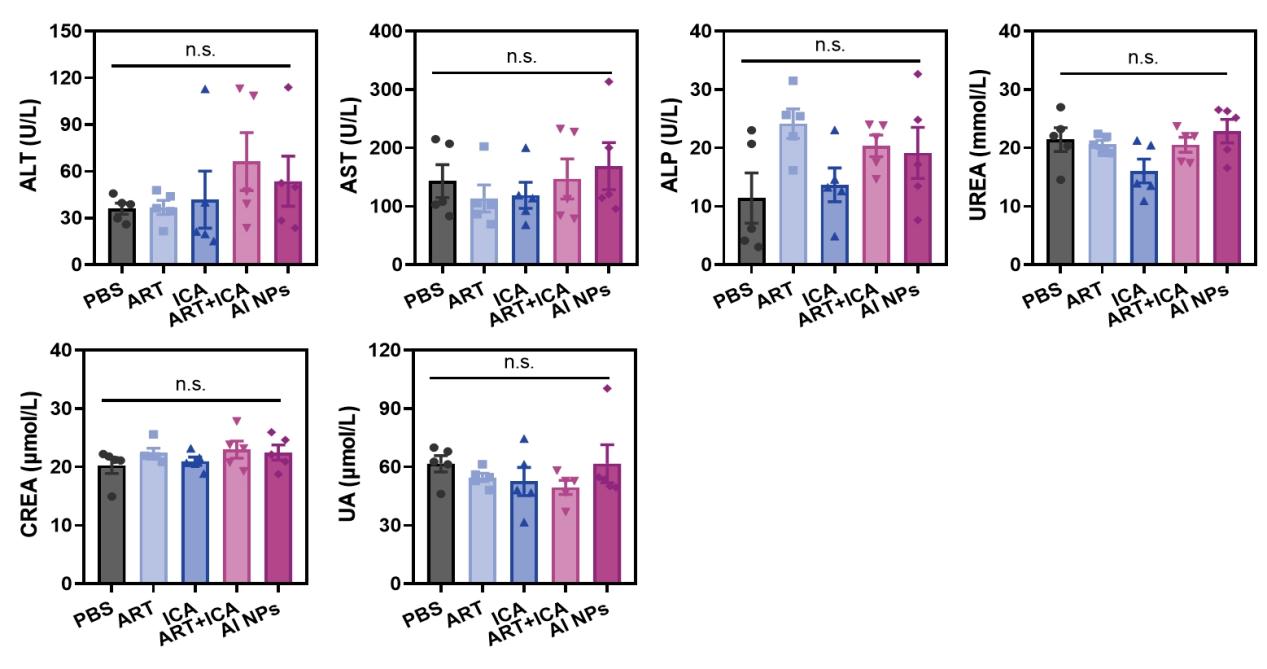


**Fig. S10.** Detection of liver and kidney function-related indicators in Hepa1-6 tumor-bearing mice after treatment with different therapeutic groups. Data are presented as mean ± S.D. (n = 5). n.s., not significant. One-way Anova.


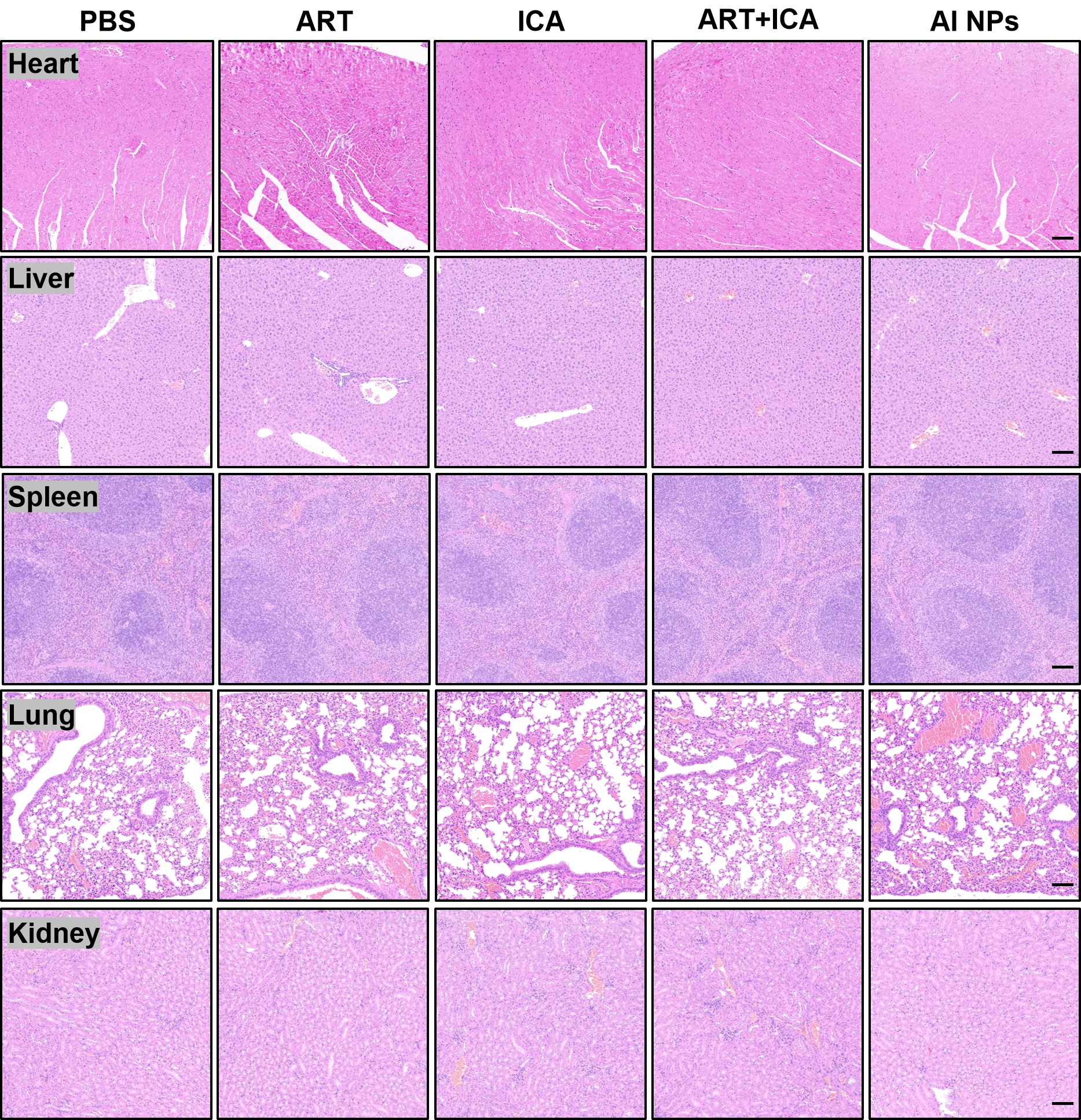


**Fig. S11.** H&E staining for major organs (heart, liver, spleen, lung, kidney) in each group. Scale bars: 100 μm.


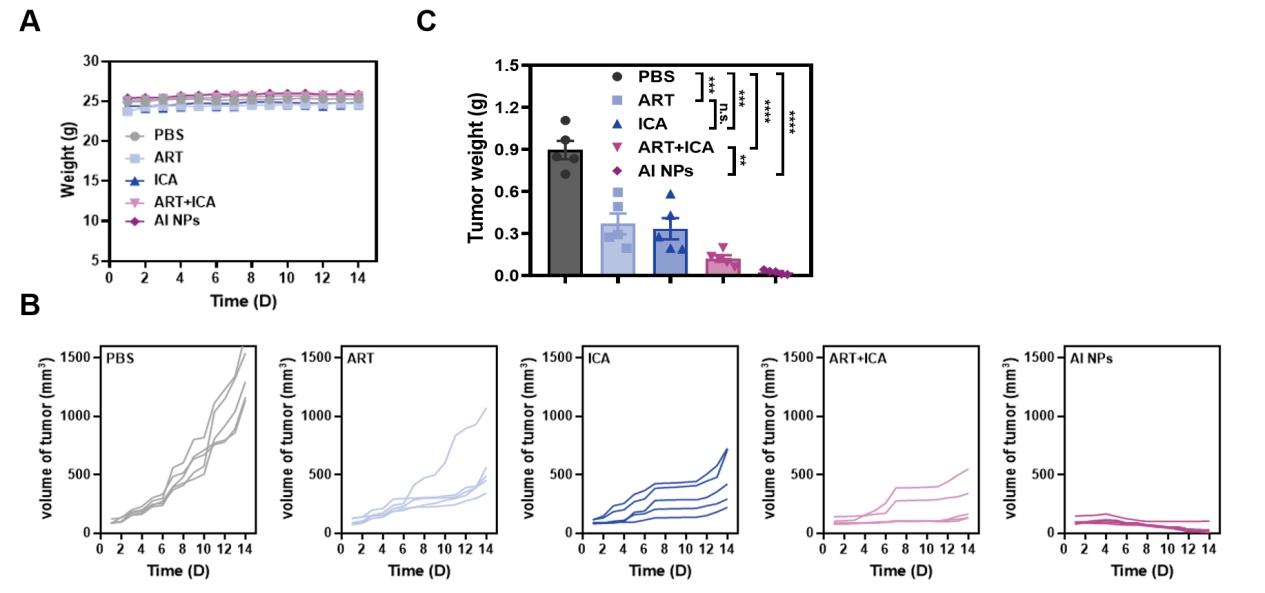


**Fig. S12.** (A) Body weight monitoring of Hepa1-6 tumor-bearing mice under treatment with different therapeutic groups. Data are presented as mean ± S.D. (n = 5). (B) Tumor growth curves for each mouse during treatment with different therapeutic groups.(C) Tumor weight of mice after treatment with different therapeutic groups. Data are presented as mean ± S.D. (n = 5). *p<0.05; **p<0.01; ***p<0.001; ****p<0.0001; n.s., not significant. One-way Anova.


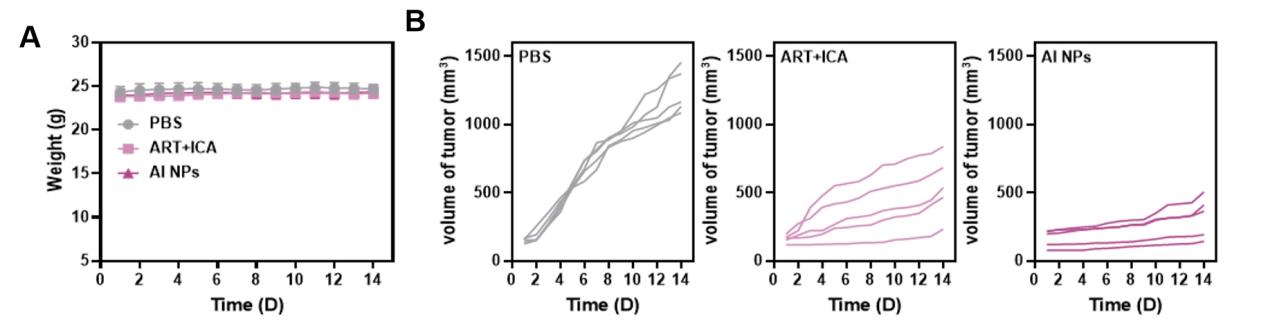


**Fig. S13.** (A) Body weight monitoring of Huh1 tumor-bearing mice under treatment with different therapeutic groups. Data are presented as mean ± S.D. (n = 5). (B) Tumor growth curves for each mouse during treatment with different therapeutic groups.


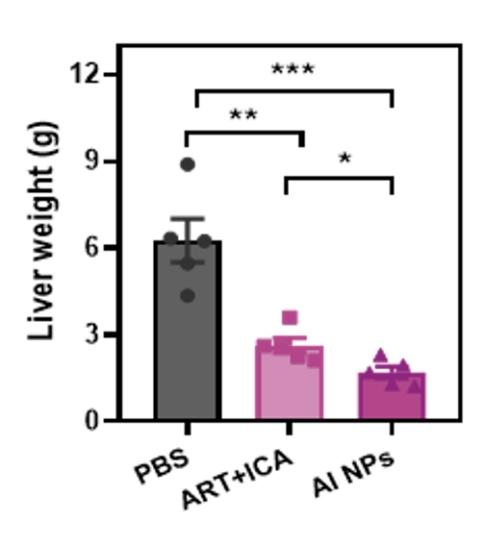


**Fig. S14.** Liver weight of Alb-tag^+^ mice after treatment with different therapeutic groups. Data are presented as mean ± S.D. (n = 5). *p<0.05; **p<0.01; ***p<0.001. One-way Anova.


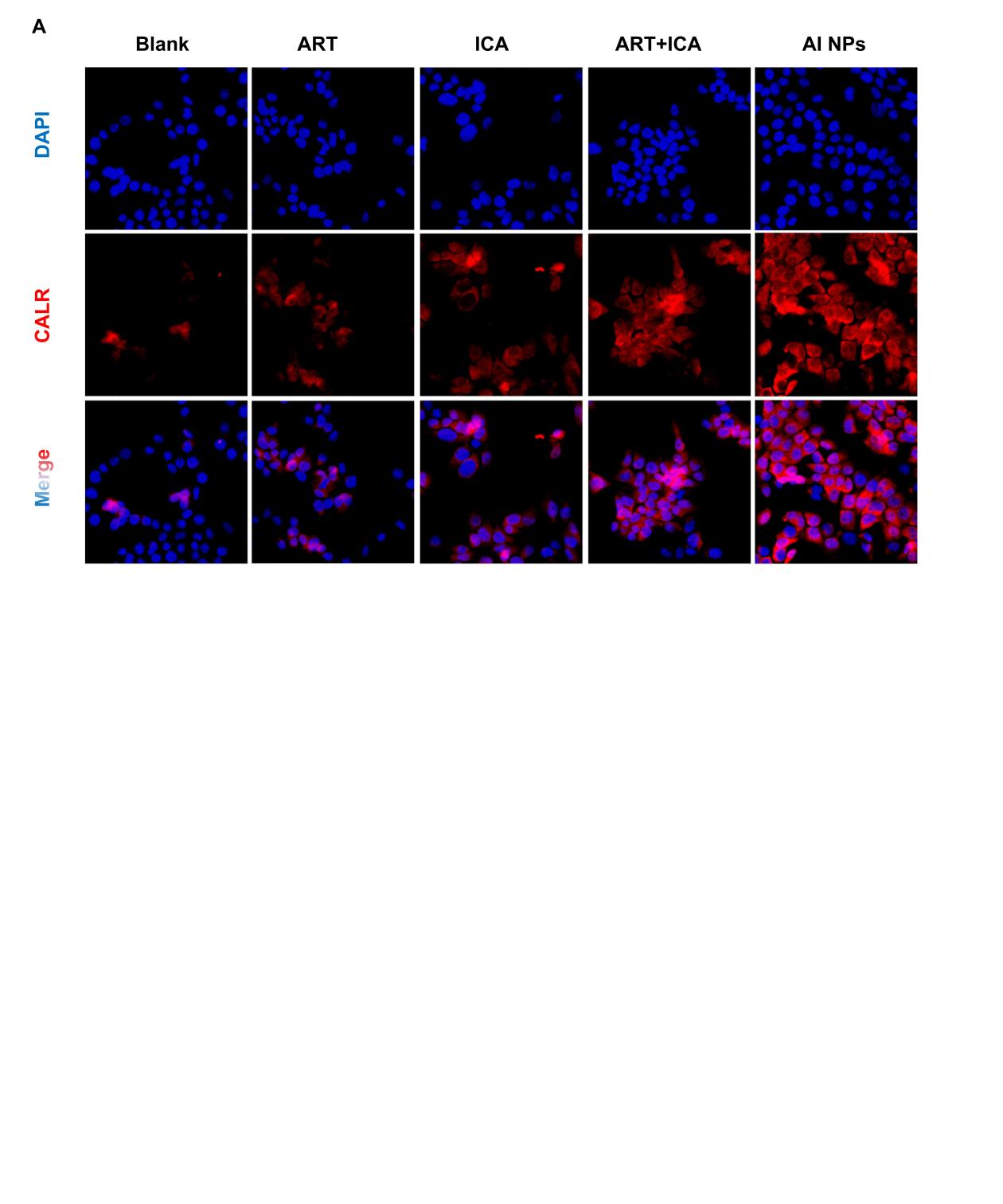


**Fig. S15.** Fluorescence staining results of CALR expression on the surface of Hepa1-6 cells after treatment with different drug formulations.


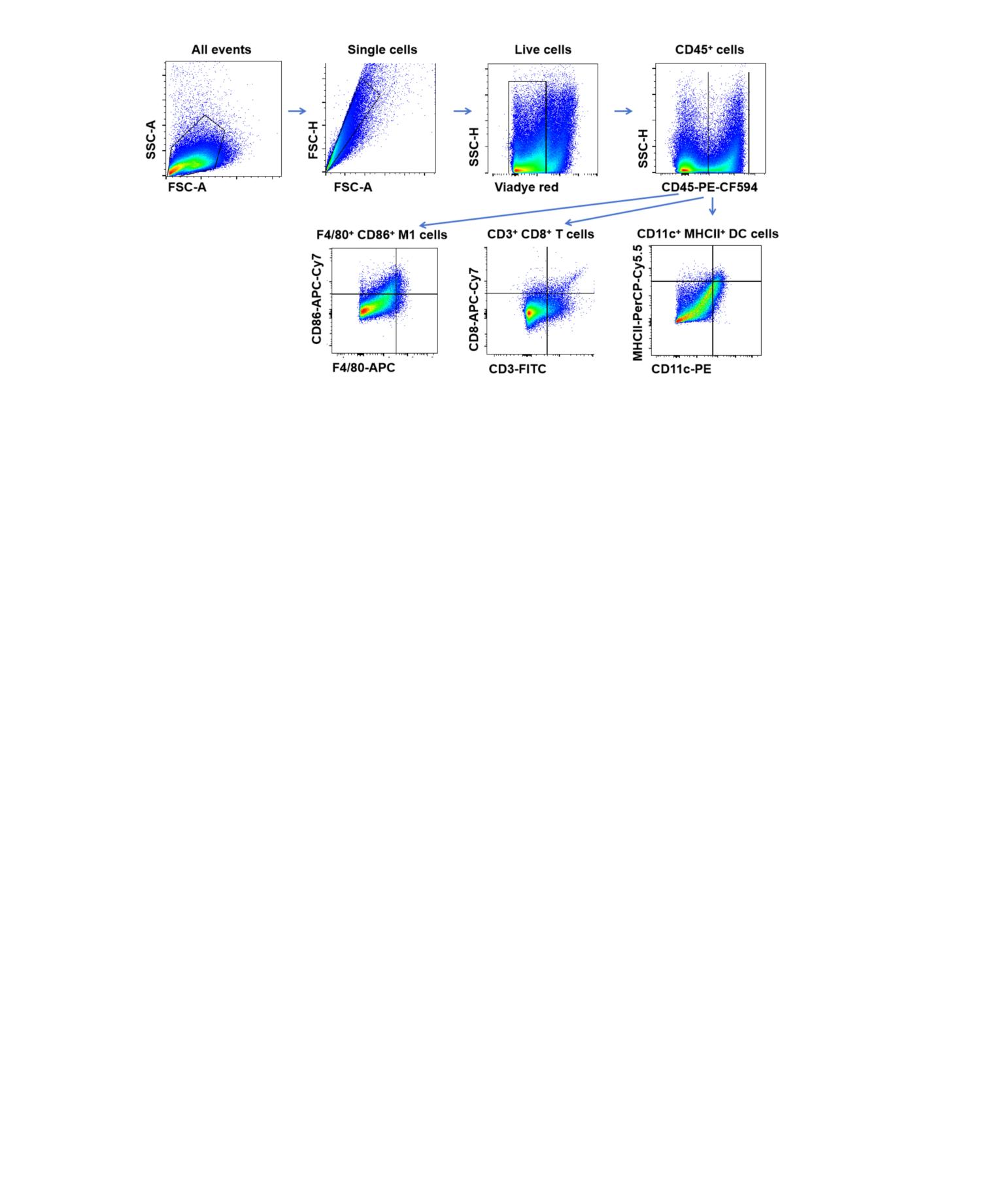


**Fig. S16.** The gating process for the flow cytometry results of Hepa1-6 tumor.


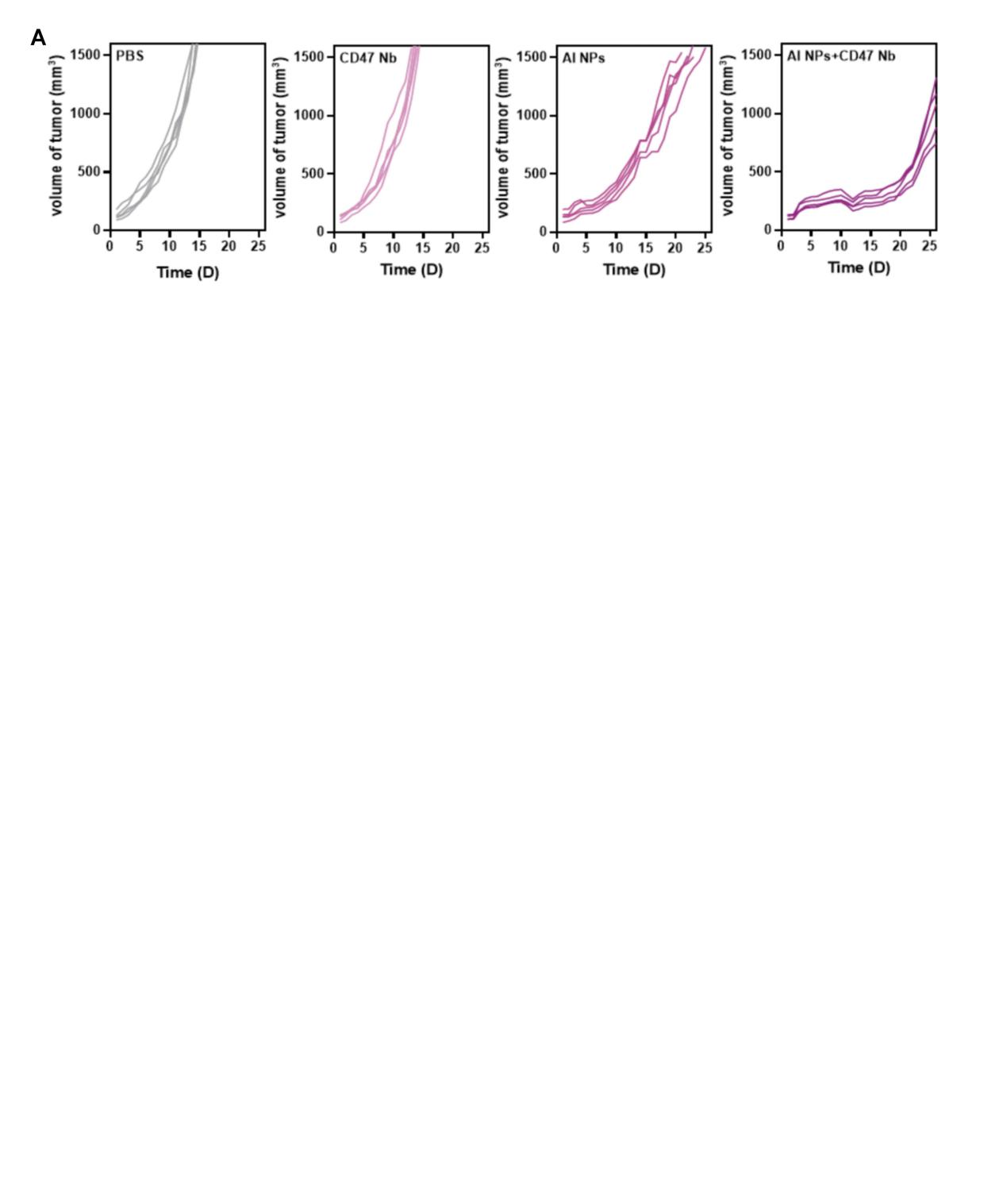


**Fig. S17.** (A) Tumor growth curves for each mouse during treatment with different therapeutic groups.
